# The central role of a two‐way positive feedback pathway in molecular targeted therapies‐mediated pyroptosis in anaplastic thyroid cancer

**DOI:** 10.1002/ctm2.727

**Published:** 2022-02-20

**Authors:** Qiwu Zhao, Haoran Feng, Zheyu Yang, Juyong Liang, Zhijian Jin, Lingxie Chen, Ling Zhan, Ming Xuan, Jiqi Yan, Jie Kuang, Xi Cheng, Ren Zhao, Weihua Qiu

**Affiliations:** ^1^ Department of General Surgery Ruijin Hospital Shanghai Jiao Tong University School of Medicine Shanghai China; ^2^ Department of General Surgery Ruijin Hospital Gubei Campus Shanghai Jiao Tong University School of Medicine Shanghai China

**Keywords:** anaplastic thyroid cancer, apatinib, caspases, melittin, pyroptosis, two‐way positive feedback

## Abstract

**Background:**

Anaplastic thyroid carcinoma (ATC) is one of the most aggressive tumours. We previously confirmed that apatinib has potential therapeutic effects on ATC via regulated cell death (RCD). As a newly identified RCD, pyroptosis demonstrates direct antitumour activity different from apoptosis or autophagy. Therefore, the clinical significance, regulatory role and underlying mechanisms of pyroptosis in ATC were focused on in this study.

**Methods:**

In a phase II trial, patients with anaplastic or poorly differentiated thyroid carcinoma received apatinib 500 mg once daily. Multiple assays were implemented to evaluate the antitumour efficacy of apatinib and/or melittin in vitro and in vivo. High‐throughput sequencing was applied to analyse differential mRNAs expression in ATC cells treated by apatinib with or without melittin. In situ Hoechst 33342/PI double‐staining, LDH release assay and enzyme‐linked immunosorbent assay (ELISA) were employed to determine pyroptosis. In mechanism exploration, quantitative RT‐PCR, Western blotting and si‐RNA knocking down were executed.

**Results:**

Seventeen patients were evaluable. Apatinib showed a promising therapeutic effect by a disease control rate (DCR) of 88.2%; however, treatment was terminated in 23.5% of patients due to intolerable toxicity. To reduce adverse events, a pyroptosis‐mediated synergistic antitumour effect of apatinib and melittin was identified in treatment of ATC in vitro and in vivo. The caspase‐1–gasdermin D (GSDMD) axis‐mediated pyroptosis was the key to extra antitumour effect of the combination of apatinib and melittin. Moreover, caspase‐3–gasdermin E (GSDME) pyroptosis pathway also functioned importantly in addition to caspase‐1–GSDMD pathway. Evidenced by in vitro and in vivo study, a two‐way positive feedback interaction was innovatively confirmed between caspase‐1–GSDMD and caspase‐3–GSDME axes.

**Conclusions:**

Through pyroptosis mediated by caspase‐1–GSDMD and caspase‐3–GSDME axes synchronically, low‐dosage apatinib and melittin could synergistically achieve a comparable therapeutic potential with reduced AEs. More importantly, a two‐way positive feedback interaction is innovatively proposed between these two axes, which provide a new prospect of targeted therapy.

## Graphical abstract

1. Apatinib and melittin could synergistically achieve a comparable therapeutic potential, which could reduce adverse events.
2. A novel antitumour effect of pyroptosis induced by combination of apatinib and melittin in anaplastic thyroid carcinoma (ATC) was identified.
3. Both caspase‐1–GSDMD and caspase‐3–GSDME pyroptosis pathways were the key to this extra antitumour effect.
4. A two‐way positive feedback regulation was innovatively proposed between caspase‐1–GSDMD and caspase‐3–GSDME axes.

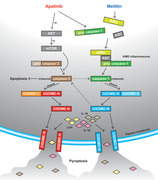

## 1 BACKGROUND

The definition of programmed cell death (PCD) has been modified recently and regulated cell death (RCD) is used to pinpoint the cell death patterns regulated by different molecular mechanisms.^1^ In this scenario, PCD is only referred to cell death in fully physiological forms.^2^ Apoptosis, necroptosis, ferroptosis, autophagy‐dependent cell death and other death patterns driven by the intracellular or extracellular perturbations constitute the rest field of RCD.

As a newly identified RCD, pyroptosis is critically defined as the formation of cytomembrane pores as a result of activation of the gasdermin family. Characterised cell death pattern of pyroptosis also includes cell swelling, damage of plasma membrane and substantial leakage of cytoplasm, especially IL‐1β.^1^, ^3^ Pyroptosis classically stems from gasdermin D (GSDMD) proteolytic cleavage as the consequence of inflammatory caspase activation.^1^, ^3^, ^4^ Recently, secondary pyroptosis has been proposed as the result of gasdermin E (GSDME/DFNA5) cleavage.^5^, ^6^ It could be elicited by multiple stimuli, including tumour necrosis factors (TNF), DNA‐damaging chemotherapeutics and infection from certain viruses. Although activated GSDMD and GSDME proteins share similar pore‐forming activities, they function individually downstream of inflammatory and apoptotic caspases. The similar function via different pathways between GSDMD and GSDME may constitute a surrogate for pyroptotic RCD, which may be crucial for the differential GSDMD and GSDME expression in different cell types and tissues under different stress status. Theoretically, most cancer cells express little GSDME,^6^ which rationalises the possible mechanism to survive from secondary pyroptosis triggered by apoptotic caspases. However, in multiple cancers, pyroptosis has been found activated easily by exogenous agents and demonstrated direct antitumour activity.^7^ Therefore, although innate resistance to apoptosis can be found in many cancers, pyroptosis induction by inflammatory and apoptotic caspases may enhance therapeutic effect of antitumour agents.

Anaplastic thyroid carcinoma (ATC), as well as poorly differentiated thyroid carcinoma (PDTC), is a sort of rare, but extremely aggressive tumour with poor prognosis and insensitivity to radiotherapy and chemotherapy.^8^ Only targeted therapy may have optimal therapeutic effect on this type of tumours.^9^ Antitumour effects of targeted therapy are generally exerted through apoptosis, autophagy, necroptosis and ferroptosis.^10^, ^11^, ^12^, ^13^ Inflammatory GSDMD‐mediated pyroptosis of macrophages activated by sorafenib targeted therapy could cause antitumour cytotoxicity of NK cells in hepatocellular carcinoma.^14^ Meanwhile, targeted regimen could trigger tumour cell pyroptosis through caspase‐3–GSDME axis in melanoma or lung cancer.^15^, ^16^ Therefore, both the types of pyroptosis have been proven to participate in antitumour effects of targeted therapy.

Apatinib, a specific inhibitor of vascular endothelial growth factor receptor 2 (VEGFR2), has been shown to induce apoptosis, autophagy and proliferation inhibition in multiple cancers.^17^, ^18^, ^19^ Melittin is an active substance isolated from bee venom. It is a water‐soluble peptide and has been used to treat chronic inflammation and various malignant tumours.^20^, ^21^, ^22^, ^23^ In this study, a pyroptosis‐mediated synergistic antitumour effect of apatinib combined with melittin was identified on the treatment of ATC in vitro and in vivo. At the mechanism level, both caspase‐1–GSDMD and caspase‐3–GSDME pyroptosis were the key to this synergistic antitumour effect. More importantly, a two‐way positive feedback interaction is innovatively proposed between caspase‐1–GSDMD and caspase‐3–GSDME axes.

## 2 METHODS

### 2.1 Patient recruitment

A prospective, single‐centre, single‐arm exploratory phase II clinical trial was employed to estimate the safety and effectiveness of apatinib in patients with ATC or advanced PDTC. The clinical study was approved by the ethics committee of Ruijin Hospital. The approval number of this clinical trial is 2017 no. 204, and this study has been registered at Chinese Clinical Trails Registry (registration no. ChiCTR2100047833). All patients signed informed consents before being enrolled. This clinical trial recruited adult patients with cytologically or histologically confirmed ATC or PDTC. The enrolment criteria of this study included at least one measurable progressive lesion in the past 3 months according to the Response Evaluation Criteria in Solid Tumors (RECIST, v1.1),^24^ and no history of prior TKI therapy or chemotherapeutics.

### 2.2 Clinical trial design, tumour response and adverse events (AEs) assessments

**FIGURE 1:**
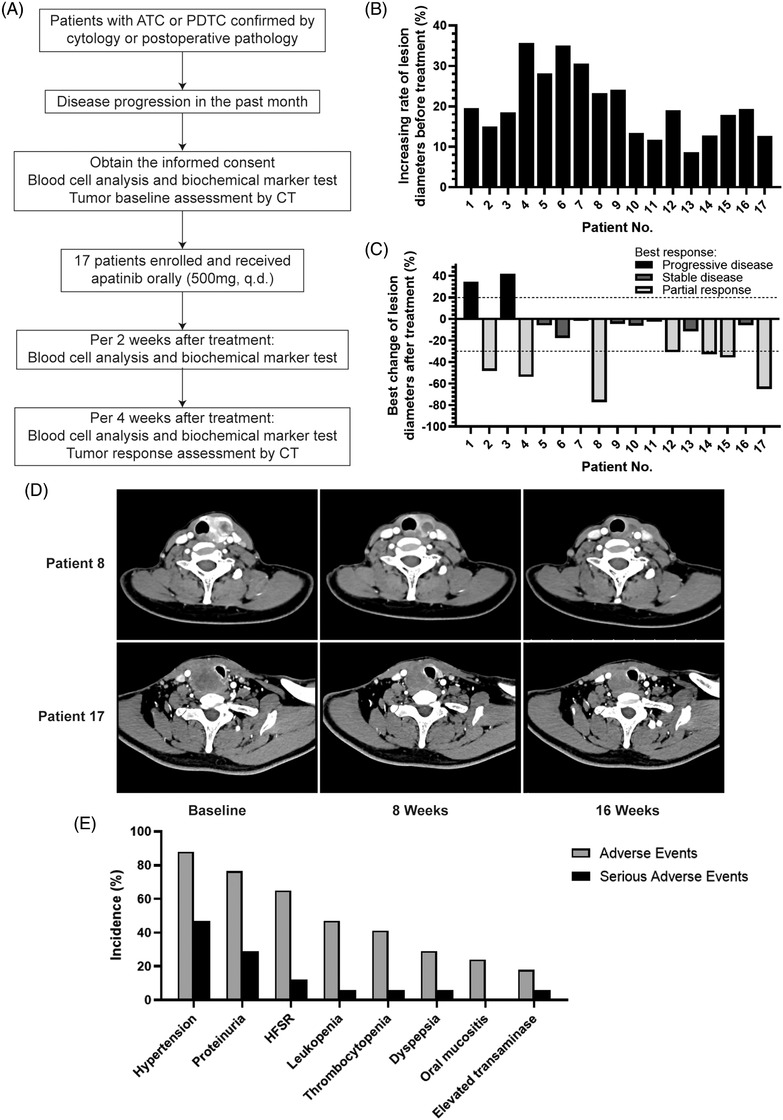
Apatinib had promising therapeutic effects and apparent AEs in ATC and PDTC treatment. (A) Schematic diagram of the phase II trial. (B) Increasing rate of the sum of target lesion diameter per month in individual patients before apatinib treatment. (C) Maximum percentage change from baseline in the sum of target lesion diameter in individual patients during apatinib treatment. Best tumour response was determined according to RECIST v1.1. (D) Computed tomography scans of two representative patients with anaplastic thyroid carcinoma collected at baseline, after 8 weeks, and after 16 weeks of treatment with apatinib. The size of their lesions was visibly reduced after 16 weeks of treatment. (E) The incidence of adverse events and serious adverse events (grade 3 and above)

Both safety and effectiveness of apatinib were evaluated. The schematic diagram of this trial is shown in Figure 1A. All patients received oral treatment with apatinib mesylate at a dosage of 500 mg quad dosage (qd). Four weeks was defined as a cycle of treatment. During the treatment, it was allowed to reduce the dosage to 250 mg qd due to AEs, but it was not allowed to increase the dosage once reduced. In a treatment cycle, interruption caused by AEs needed to be less than three times, and the cumulative time needed to be less than 2 weeks. The treatment was terminated when drug became intolerant, disease progressed or patient voluntarily withdrew from this study. The primary endpoint of this study was disease control rate (DCR). The secondary endpoints included objective response rate (ORR) and safety. Blood cell analysis and biochemical marker test were performed before the treatment and every 2 weeks during the treatment. Tumour diameters (TD) were measured by computed tomography before the treatment and every 4 weeks during the treatment. Relative tumour diameter was calculated as (TD/TD_baseline_), and tumour response was assessed according to RECIST v1.1. The evaluation of AEs continued until at least 4 weeks after the last dose.

### 2.3 Cell culture and treatments

Human ATC cell lines CAL‐62 and C‐643 were purchased from the China Center for Type Culture Collection. CAL‐62 and C‐643 cells were cultured in DMEM and RPMI‐1640 medium supplemented with 10% fetal bovine serum (Gibco, USA) at 37°C in 5% CO_2_, respectively. Apatinib mesylate (YN968D1), VX‐765 and Z‐DEVD‐FMK were purchased from TargetMol (Target Molecule Corp, USA). These agents were first dissolved in DMSO and then diluted with DMEM or RPMI‐1640 medium with a final DMSO concentration of less than 0.4% for in vitro studies. Melittin was purchased from Sigma‐Aldrich and was first dissolved in PBS and then diluted with DMEM or RPMI‐1640. N‐Acetyl‐L‐cysteine (NAC) was purchased from Beyotime (China). All cells were cultured in well plate for at least 24 h before each treatment.

### 2.4 Cell viability assay

Cell counting kit‐8 (CCK‐8, Meilunbio, China) was used to determine cell viability. In short, DMEM or RPMI‐1640 medium containing apatinib and/or melittin was added to 96‐well plate seeded with ATC cells. Cells were incubated with CCK‐8 working solution for 1–2 h after treatment, and then OD450 value was measured using a microplate reader (Epoch, BioTek). The combined effects of the drugs were evaluated using the Chou–Talalay method.^25^

### 2.5 Cell proliferation assay

EdU incorporation assay kit (C0075L, Beyotime) was applied to determine cell proliferation rate. In short, DMEM or RPMI‐1640 medium containing apatinib and/or melittin was added to six‐well plate seeded with ATC cells. Cells were incubated with EdU working solution for 2 h after treatment, and then fixed with 4% paraformaldehyde. Subsequently, Azide 555 was added to the above cells treated with 0.5% Triton X100 and incubated for 30 min. Then cells were stained with Hoechst 33342. Finally, the percentage of fluorescence‐positive cells was calculated to assess the rate of proliferation.

Colony formation assay was employed as well. DMEM or RPMI‐1640 medium containing apatinib and/or melittin was added to six‐well plate seeded with low‐density ATC cells for 7 days. The colonies were stained with 0.1% crystal violet methanol solution for observation.

### 2.6 Xenograft tumour model

As previously described,^26^ 6–8‐week‐old male BALB/c nude mice were purchased from Institute of Zoology, Chinese Academy of Sciences (Shanghai, China). Subcutaneous injection of CAL‐62 cells in mice was operated to establish xenograft models, and each injection was 100 μl of PBS containing 1 × 10^7^ cells. All mice were divided into eight groups (group 1: vehicle‐only solution; group 2: low‐dose apatinib 25 mg/kg; group 3: standard‐dose apatinib 50 mg/kg; group 4: low‐dose apatinib 25 mg/kg + melittin 1 mg/kg; group 5: melittin 1 mg/kg; group 6: low‐dose apatinib 25 mg/kg + melittin 1 mg/kg + VX‐765 100 mg/kg; group 7: low‐dose apatinib 25 mg/kg + melittin 1 mg/kg + Z‐DEVD‐FMK 2 mg/kg; and group 8: low‐dose apatinib 25 mg/kg + melittin 1 mg/kg + VX‐765 100 mg/kg + Z‐DEVD‐FMK 2 mg/kg). Apatinib, was first dissolved in DMSO and then diluted with NS (normal saline solution), was administered by gavage daily. Melittin, dissolved in ddH_2_O, was intraperitoneally injected each day. VX‐765 and Z‐DEVD‐FMK, dissolved in 0.5% hydroxyethyl cellulose aqueous solution containing 0.1% Tween‐80, were injected intraperitoneally daily. The length and width of tumour nodules were measured with a vernier caliper every week, and volume of tumour nodules was estimated according to the formula of *V* = *π*/6 × (*W* ^2^ × *L*). On the fourth week after establishment of xenograft models, the mice were killed by cervical dislocation and the tumour nodules were removed, weighed and fixed in formalin. The liver and kidneys of the mice were also removed and fixed in formalin.

Immunohistochemical (IHC) staining according to previous description was performed subsequently.^26^ Antibodies included anti‐Ki67 (Santa Cruz, sc‐23900), anti‐CD31 (CST, 3528), anti‐IL‐1β (ABclonal, A16288), anti‐IL‐18 (ABclonal, A1115), anti‐AIM2 (abcam, ab93015), anti‐cleaved caspase‐1 (CST, 4199), anti‐GSDMD‐NT (CST, 36425), anti‐cleaved caspase‐3 (CST, 9664) and anti‐GSDME‐NT (abcam, ab222408). In Situ Cell Death Detection Kit (Roche Applied Science, USA) was applied to conduct TUNEL assay. DAPI was used to stain the nucleus. The immunohistochemical score was determined based on the proportion of positive cells and the positive intensity.

### 2.7 RNA‐seq analyses

RNA was extracted from CAL‐62 cells treated with 20 μM apatinib, with or without 4 μg/ml melittin, for 24 h. After passing RNA quality control, Ion Total RNA‐Seq Kit v2 (Thermo Fisher, 4479789) was used to prepare the sequencing library according to manufacturer's instructions. After performing emulsion PCR on the library template above, the template‐positive Ion Sphere particles were enriched. Subsequently, Ion PITM chip was employed for the following sequencing. The raw data were standardised, followed by further analysis using Agilent GeneSpring GX software. Differentially expressed genes (DEGs) were determined according to the fold change of expression (fold change not less than 2) and statistical significance (*p* < .05). Finally, Kyoto Encyclopedia of Genes and Genomes (KEGG) analysis and Gene Ontology (GO) functional enrichment were employed to identify the roles of DEGs. Gene set enrichment analysis (GSEA) was conducted to analyse differentially expressed gene sets.

### 2.8 Hoechst 33342 and propidium iodide staining

To monitor the plasma membrane integrity, Hoechst 33342 and propidium iodide (PI) double staining was performed. In short, DMEM or RPMI‐1640 medium containing indicated agents was added to six‐well plate seeded with ATC cells. The cells were incubated with Hoechst 33342 (1 mg/ml) and PI (5 mg/ml) for 15 min at 37°C. A Nikon TS2R‐FL was employed to excite fluorescence and take typical pictures, and Image J software was then applied to quantify the proportion of positive cells.

### 2.9 LDH release assay

To confirm that cells underwent pyroptosis rather than apoptosis, we used LDH release as an indicator of cell death. In short, DMEM or RPMI‐1640 medium containing indicated agents was added to six‐well plate seeded with ATC cells. The culture supernatant was centrifuged at 400 × *g* for 5 min after treatment and then loaded into a 96‐well plate. LDH release assay kit (Beyotime, C0017) was employed for subsequent detection. The LDH release level of cells above was assessed according to the formula (ODR_sp_ − ODR_bg_)/(ODR_max_ − ODR_bg_) × 100%, of which ODR_sp_, ODR_bg_ and ODR_max_ were the OD490/OD600 ratio value for sample culture supernatants, medium and cell lysates, respectively.

### 2.10 ELISA

Enzyme‐linked immunosorbent assay (ELISA) kits (Merck, RAB0273 and RAB0543) were employed to assess the influence of apatinib and/or melittin on IL‐1β and IL‐18 production. In short, DMEM or RPMI‐1640 medium containing indicated agents was added to six‐well plate seeded with ATC cells. The culture supernatant was centrifuged at 400 × *g* for 5 min after treatment and then loaded into a 96‐well plate. Antibodies, enzymes and substrates were added to sample wells in order and incubated according to the manufacturer's instruction. After terminating the reaction with stop solution, OD450 value was measured using a microplate reader (Epoch, BioTek).

### 2.11 RNA extraction and quantitative RT‐PCR

TRIzol Reagent (Invitrogen, 15596018) was used to extract total RNA, and HiScript III RT SuperMix (Vazyme, R323‐01) was employed to perform reverse transcription reaction. Quantitative RT‐PCR (qPCR) experiments were conducted with ChamQ SYBR Color qPCR Master Mix (Vazyme, Q431‐02). In short, the cycle program of qPCR consisted of denaturation (95°C, 15 s), annealing (60°C, 15 s) and extension (72°C, 45 s). Raw data were processed with the 2^–ΔΔCt^ method. Sequences of primer pairs used in this study are shown in Table S1.

### 2.12 Western blotting

As previously described,^26^ Laemmli sample buffer (BIO‐RAD, 1610737), containing ProtLytic protease and phosphatase inhibitor cocktail (New Cell & Molecular Biotech, P002) and 5% 2‐mercaptoethanol, was added to cells after indicated treatment and then boiled. Per well, 30–50 μg protein was loaded to 10% or 12.5% SDS–PAGE gel; and first separated by electrophoresis and then cross‐linked to PVDF membrane by wet electro‐transfer system. TBS‐T containing 3% BSA was applied to block the blank sites on the membrane. The primary antibodies used in this study included anti‐GAPDH (abcam, ab8245), anti‐caspase‐1 (CST, 3866), anti‐cleaved caspase‐1 (ABclonal, A16792), anti‐GSDMD (CST, 97558), anti‐GSDMD‐NT (ABclonal, A10164), anti‐AIM2 (CST, 12948), anti‐NLRP7 (ABclonal, A11627), anti‐caspase‐3 (abcam, ab32150), anti‐cleaved caspase‐3 (CST, 9664), anti‐GSDME (ABclonal, A7432), anti‐GSDME‐NT (abcam, ab215191), anti‐caspase‐8 (abcam, ab32397), anti‐cleaved caspase‐8 (CST, 9496), anti‐phospho‐MLKL (ABclonal, AP0949), anti‐MLKL (ABclonal, A5579) and anti‐caspase‐4 (MBL, M029‐3). Secondary antibodies were HRP‐conjugated Affinipure Goat Anti‐Mouse IgG (H+L) (Proteintech, SA00001‐1) and HRP‐conjugated Affinipure Goat Anti‐Rabbit IgG (H+L) (Proteintech, SA00001‐2). Enhanced chemiluminescence detection system (Tanon, 4600) was employed for visualising and imaging. GAPDH was applied as internal control.

### 2.13 Transfection of si‐RNA

The AIM2, NLRP7, GSDMD and GSDME‐targeting siRNAs were ordered from Bioegene (Shanghai, China). The sequences were as follows: si‐AIM2 sense: GGAACAAUUGUGAAUGGUUTT, si‐AIM2 antisense: AACCAUUCACAAUUGUUCCTT, si‐NLRP7 sense: GACGUCACUCUGAGAAACCAATT, si‐NLRP7 antisense: UUGGUUUCUCAGAGUGACGUCTT, si‐GSDMD sense: CCUUCUCUUCCCGGAUAAGAATT, si‐GSDMD antisense: UUCUUAUCCGGGAAGAGAAGGTT, si‐GSDME sense: GCAUGAUGAAUGACCUGACUUTT and si‐GSDME antisense: AAGUCAGGUCAUUCAUCAUGCTT. Lipofectamine 3000 transfection reagent (Thermo Fisher, L3000015) was applied for transient transfection of CAL‐62 and C‐643 cells according to manufacturer's instruction.

### 2.14 Reactive oxygen species assay

Reactive oxygen species (ROS) assay kit (Beyotime, S0033) was applied for intracellular ROS detection. In short, DMEM or RPMI‐1640 medium containing apatinib and/or melittin was added to six‐well plate seeded with ATC cells. DCFH‐DA working solution was added to cells after treatment and incubated for 20 min. A FACSCalibur flow cytometer (Becton–Dickinson) was employed for fluorescence intensity detection.

### 2.15 CRISPR–Cas9 knockout cell lines

Caspase‐1 gRNA (5′‐TAATGAGAGCAAGACGTGTG‐3′) and caspase‐3 gRNA (5′‐ATGTCGATGCAGCAAACCTC‐3′) were cloned into LentiCRISPRv2, respectively, as previously described.^27^ The above plasmids were transfected into 293T cells with lentivirus helper plasmids. After 48 h, virus was concentrated from the above cell culture supernatant and applied for ATC cells infection. Puromycin (5 μg/ml) was then added into medium to select for positive cells for 3 days.

### 2.16 Statistics

The data were processed as previously described.^19^ In short, Mann–Whitney *U*‐test, ANOVA or Student's *t*‐test were employed for significance testing between groups. Fisher's exact test or *χ* ^2^ test was used to assess categorical variables. Statistical Product and Service Solutions (SPSS, IBM, v22) was employed for data processing, and *p* < .05 was considered statistically significant.

## 3 RESULT

### 3.1 Apatinib had promising therapeutic effects and apparent AEs in ATC and PDTC treatment

A total of 17 eligible patients were enrolled in the study and received apatinib 500 mg qd treatment (Figure 1A, Table 1). The average age of the patients was 62 years, of which 35.3% were female. Compared with increasing rate of the sum of lesion diameters before treatment, apatinib showed a promising therapeutic effect. The best tumour response during apatinib therapy was 41.2% for partial response (PR), 47.0% for stable disease (SD) and 11.8% for progressive disease (PD); DCR and ORR were 88.2% and 41.2%, respectively (Figure 1B,C). Representative CT images of patients during follow‐up are shown in Figure 1D.

**TABLE 1:** Baseline clinical characteristics of enrolled patients and best tumour response during treatment

Patient No.	Gender	Age	Pathology	TNM stage	Metastasis site	Treatment duration (weeks)	Best response
1	F	80	ATC	T4aN1bM1	Lung, bone	8	PD
2	F	66	PDTC	T4bN1bM1	Lung, bone, axilla	33	PR
3	F	67	ATC	TxN1aM1	Lung, bone	62	PD
4	M	63	PDTC	T3bN1bM1	Bone	11^a^	PR
5	F	80	PDTC	T4aNxM1	Lung	68	SD
6	F	57	ATC	TxN1bM1	Lung, bone	43	SD
7	F	46	PDTC	T4bN1bM1	Lung, bone	104	SD
8	F	42	ATC	TxN1bM0	–	49	PR
9	F	81	ATC	T4bN1aM0	–	52	SD
10	M	61	PDTC	T4aN1bM1	Lung, bone, adrenal gland	51	SD
11	F	66	PDTC	T3bN1bM1	Lung	87	SD
12	F	78	ATC	TxN1bM1	Lung, bone	15^a^	PR
13	M	56	PDTC	T3bN1bM1	Brain	56	SD
14	M	55	PDTC	T3aN1bM1	Lung, bone	13^a^	PR
15	M	42	ATC	TxN1bM1	Bone	73	PR
16	M	57	ATC	T4bNxM1	Lung	9^a^	SD
17	F	59	PDTC	T3bN1bM0	–	56	PR

Abbreviations: ATC, anaplastic thyroid carcinoma; PD, progressive disease; PDTC, poorly differentiated thyroid carcinoma; PR, partial response; SD, stable disease.

^a^ Treatment was terminated due to intolerable toxicity.

Treatment was terminated in 23.5% of the patients due to intolerable toxicity (grades 3 and 4 AEs). The highest incidence of AEs was hypertension, with an incidence of 88.2% (47.1% for grades 3 and above, Figure 1E). Other AEs included proteinuria (76.5% for all grades and 29.4% for grade 3 and above), hand–foot skin reaction (HFSR; 64.7% for all grades and 11.8% for grades 3 and above), leukopenia (47.1% for all grades and 5.9% for grades 3 and above), thrombocytopenia (41.2% for all grades and 5.9% for grades 3 and above), dyspepsia (29.4% for all grades and 5.9% for grades 3 and above), oral mucositis (23.5% for all grades and 0% for grades 3 and above) and elevated transaminase (17.6% for all grades and 5.9% for grades 3 and above).

### 3.2 Apatinib and melittin had a synergistic effect on the treatment of ATC in vitro and in vivo

Considering the AEs in apatinib treatment, possible mechanisms to decrease side effects of apatinib have been explored. In this study, the synergistic effect is focused on to decrease dosage of apatinib without compromising therapeutical benefits.

**FIGURE 2:**
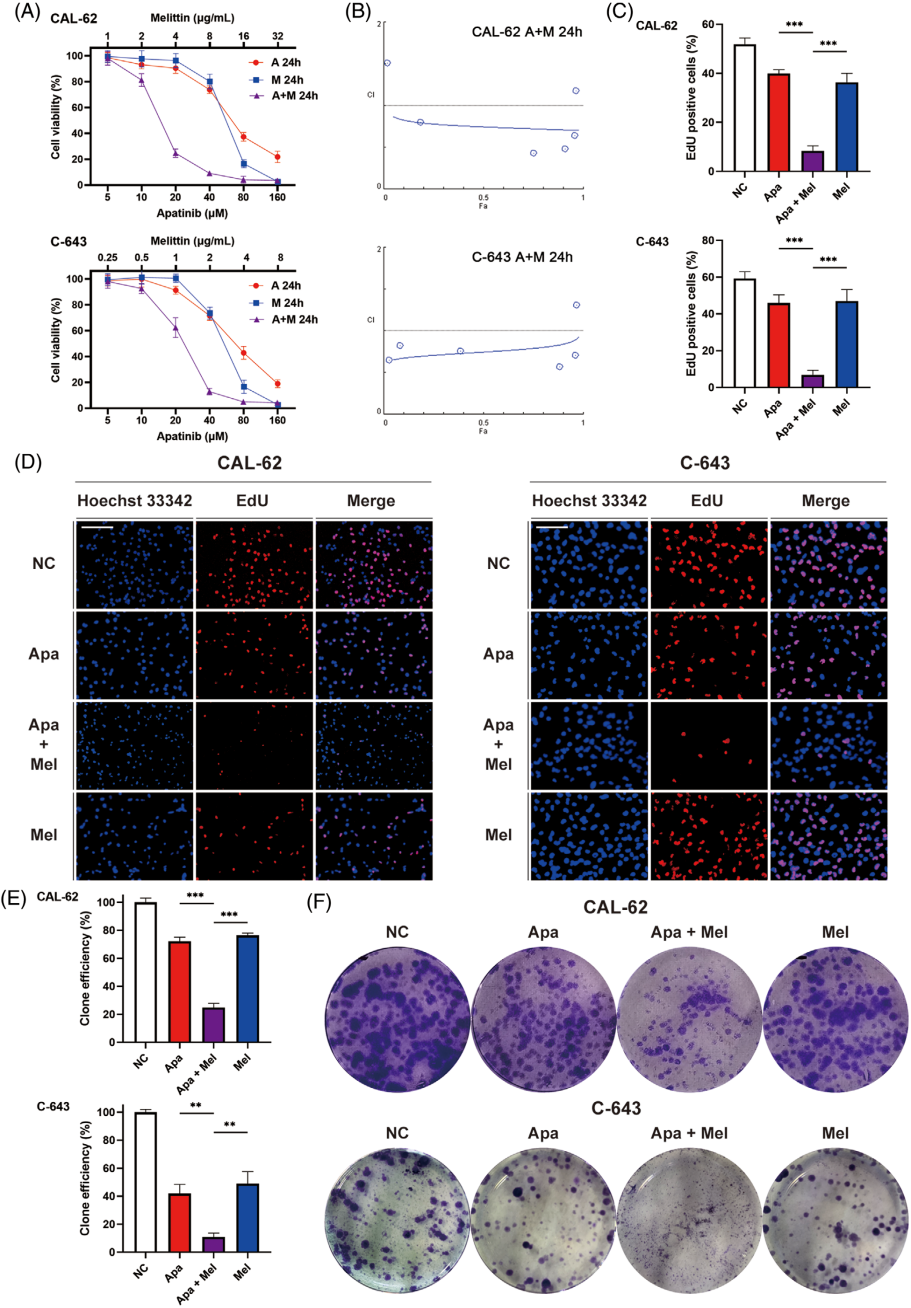
Apatinib and melittin had a synergistic effect on the treatment of ATC in vitro. (A) ATC cells were administered with ascending concentrations of apatinib and/or melittin, and cell viability was assessed by CCK‐8 assay. (A) *F*_a_‐CI plot for combined application of apatinib and melittin using the Chou–Talalay method. (C and D) Quantitative results and representative images of cell proliferation measured by EdU incorporation assay after being treated by apatinib (20 μM) and/or melittin (4 μg/ml for CAL‐62 and 1 μg/ml for C‐643) for 24 h (scale bar: 200 μm). (E and F) Quantitative results and representative images of cell proliferation measured by colony formation assay after being treated by apatinib (10 μM) and/or melittin (2 μg/ml for CAL‐62 and 0.5 μg/ml for C‐643) for 2 weeks. Data are represented as mean ± SD; *p < .05, **p < .01, ***p < .001

We first established the dose–response relationship between apatinib and/or melittin in vitro. Two ATC cell lines CAL‐62 and C‐643 were administered with ascending concentrations of apatinib with/without melittin. The IC‐50 values of apatinib for CAL‐62 and C‐643 were 66.76 and 68.75 μM for 24 h, and the IC‐50 values of for melittin for CAL‐62 and C‐643 were 10.85 and 2.58 μg/ml for 24 h, respectively (Figure 2A). The inhibitory effects of the two drugs were evaluated at different concentrations on ATC cells when used alone or in combination. The Chou–Talalay method was then employed to evaluate the synergy of the two drugs quantitatively (Figure S1A,B). The results showed that the combination index (CI) value at ED50 was about 0.75, suggesting possible synergistic effect rather than additive effect (Figure 2B and Figure S1C,D, Table S2). EdU incorporation assay was further used to detect cell proliferation. The results showed that apatinib and melittin slightly reduced the proliferation efficiency of CAL‐62 and C‐643 cells, but the combined application of the two drugs at the above concentrations can significantly inhibit the cell proliferation (Figure 2C,D). Subsequently, we used colony formation to confirm that the combined application of apatinib and melittin inhibited the proliferation of ATC cells more significantly than solo application (Figure 2E,F). Thus, synergistic effect was confirmed in vitro with the combined use of the two drugs.

**FIGURE 3:**
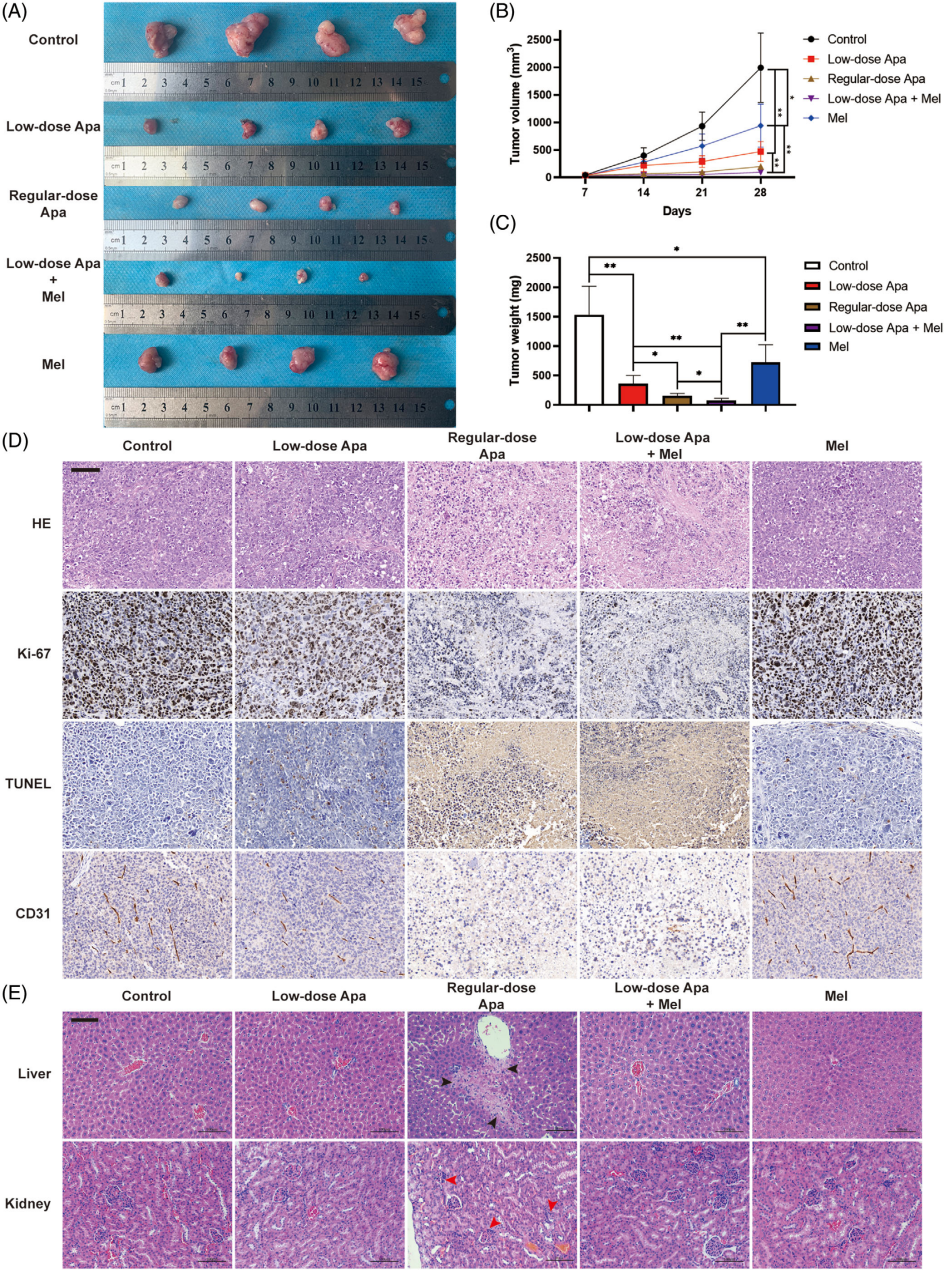
Apatinib and melittin had a synergistic effect on the treatment of ATC in vivo. (A) Tumours of xenograft mice with subcutaneous injection of CAL‐62 cells. The mice were given vehicle control, low‐dose apatinib, standard‐dose apatinib, low‐dose apatinib combined with melittin and only melittin, respectively. (B) Tumour volumes were measured every week. (C) Tumour weights were analysed after mice sacrifice. (D) Representative images of HE staining, IHC staining of Ki‐67, CD31 and TUNEL assay of tumours of indicated treatment (scale bar: 50 μm). (E) Representative images of HE staining of livers and kidneys of indicated treatment mice (scale bar: 100 μm). Black arrowheads indicate hepatocyte necrosis and lymphocyte infiltration. Red arrowheads indicate glomerular atrophy. Data are represented as mean ± SD; *p < .05, **p < .01, ***p < .001

On the basis of the results above, xenograft tumour models were further established to explore the inhibitory effect of the two drugs alone or in combination on tumour proliferation in vivo. Five groups of the mice with subcutaneous injection of CAL‐62 cells were given vehicle control, low‐dose apatinib, standard‐dose apatinib, low‐dose apatinib combined with melittin and melittin alone. The results showed that both low‐dose apatinib and melittin solo treatment can inhibit tumour proliferation by 76.3% and 52.7%, respectively. The inhibitory effect of apatinib showed dose‐dependent relationship in regular‐dose group. Meanwhile, the inhibitory effect of combined application of two drugs is more significant than solo treatment (95.2% vs. 76.3%, *p *< .01). Interestingly, low dose of apatinib in the combined application can achieve even better effect than that in regular‐dose apatinib single drug (95.2% vs. 89.9%, *p *< .05, Figure 3A–C). Histological study indicated that the combined application of two drugs resulted in stronger proliferation inhibition (Ki67‐positive rate) and more cell death (TUNEL‐positive rate, Figure 3D). In addition, CD31 staining indicated that melittin had no significant inhibitory effect on the angiogenesis of xenograft models, suggesting that antiangiogenesis did not play an important role in this synergistic antitumour effect (Figure 3D). The consistent data from in vitro and in vivo concluded that low‐dosage apatinib with melittin could achieve a comparable therapeutic potential.

More importantly, the influences of regular‐dose apatinib and the two‐drug combination on liver and kidney tissues were further portrayed. We found that regular‐dose apatinib could cause apparent liver tissue necrosis and glomerular atrophy. The low dosage of apatinib, no matter in solo treatment or combined application, caused little or no liver and kidney tissue damage, suggesting that melittin can improve the therapeutic effect of apatinib on ATC without increasing liver and kidney toxicity (Figure 3E).

Thus, the extra antitumour effect of apatinib stemmed from the synergistic interaction, which could reduce the dosage of apatinib with decreasing adverse reactions.

### 3.3 Caspase‐1–GSDMD pyroptosis pathway was the key to extra antitumour effect of the combination of apatinib and melittin

**FIGURE 4:**
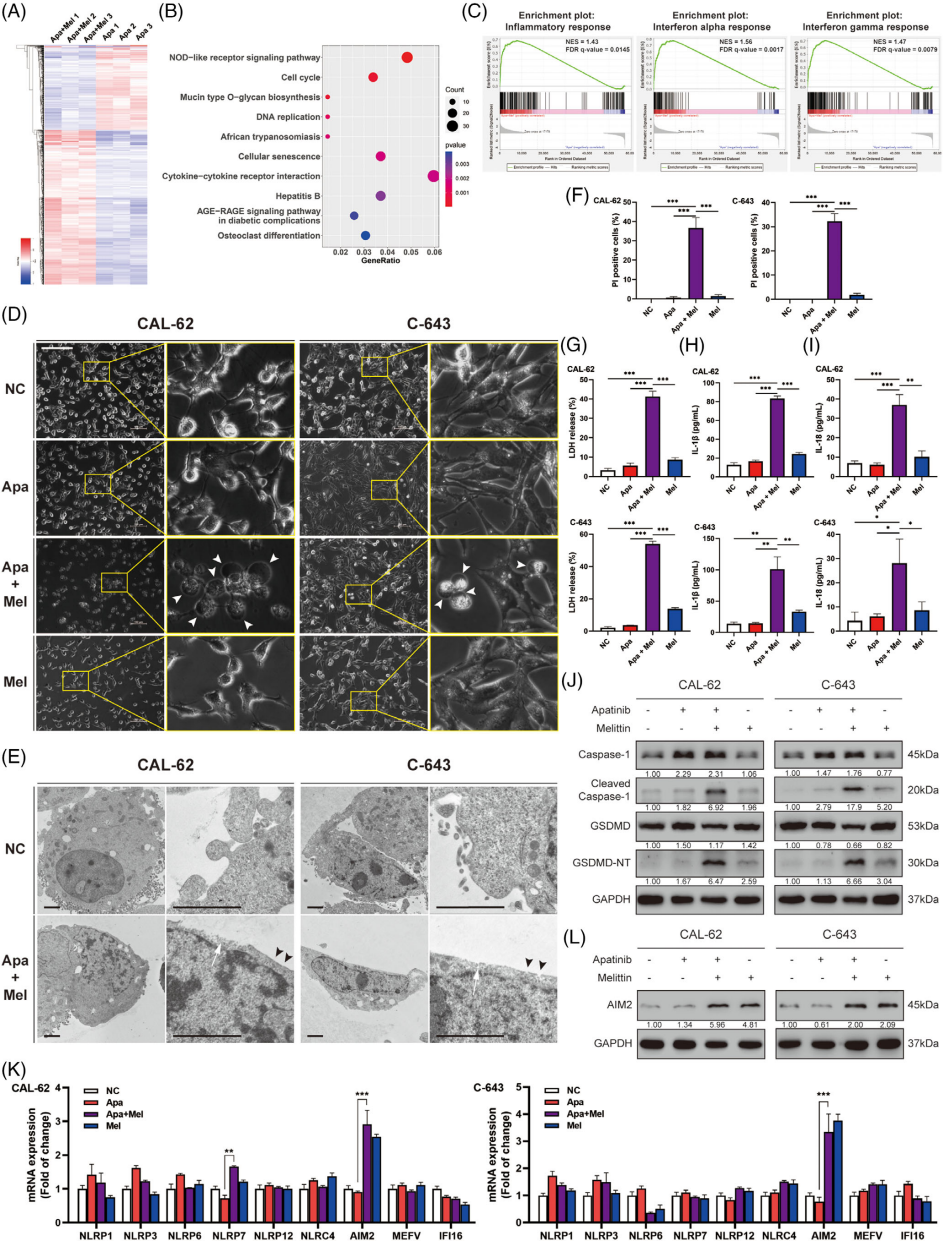
Caspase‐1–GSDMD pyroptosis pathway provided an extra antitumour effect of the combination of apatinib and melittin. (A) Heatmap showing differentially expressed mRNAs in CAL‐62 treated by apatinib with or without melittin, analysed by high‐throughput RNA‐sEquation. (B) Result of KEGG pathway analysis using RNA‐seq data. (C) Result of generation enrichment analysis (GSEA) using RNA‐seq data. (D) Representative images of cell morphology after indicated treatment taken by a phase contrast microscope (scale bar: 200 μm). White arrowheads indicate bubbles from the plasma membrane. (E) Transmission electron micrographs of ATC cells after indicated treatment. Black arrowheads indicate the membrane pores and white arrows indicate disrupted regions of membrane (scale bar: 2 μm). (F) Quantitative results of PI‐positive rate of ATC cells after indicated treatment by in situ Hoechst 33342/PI double staining. (G) LDH release level of ATC cells after indicated treatment. (H and I) IL‐1β and IL‐18 production of cells after indicated treatment. (J) Representative Western blotting images of cells after indicated treatment. The protein level of cleaved caspase‐1 and GSDMD‐N terminal were significantly increased when apatinib and melittin were used in combination. Caspase‐1 was upregulated after apatinib treatment, whether melittin was applied. (K) mRNA transcription levels of nine different NOD‐like receptors in cells after indicated treatment measured by qPCR. (L) AIM2 protein level was upregulated after melittin treatment, whether apatinib was applied. ATC cells were treated by apatinib (20 μM) and/or melittin (4 μg/ml for CAL‐62 and 1 μg/ml for C‐643) for 24 h for all assays. Data are represented as mean ± SD; *p < .05, **p < .01, ***p < .001

We next performed RNA‐seq analyses to unravel molecular mechanisms of melittin enhancing the antitumour effect of apatinib. Clustering analysis was employed for visualising the fold change of expression profiles between the two‐drug combined treated CAL‐62 cells group and the apatinib single‐drug treatment group (Figure 4A). According to the sequencing results, 214 DEGs were identified and annotated by KEGG analysis. Pathway analysis was conducted subsequently. The results demonstrated that the most significant upregulated pathway in the two‐drug combined group was NOD‐like receptor signalling pathway (Figure 4B). Meanwhile, the results of GO molecular function enrichment showed that melittin significantly increased cytokine activity (Figure S2A). In addition, GSEA showed that several datasets were enriched in the dual‐drug combination group. Among these, three (interferon alpha response datasets, interferon gamma response datasets and inflammatory response datasets) were closely related to the NOD‐like receptor signalling pathways. Thus, melittin may have an impact on inflammasomes in ATC cells with presence of apatinib (Figure 4C).

By the phase‐contrast microscope, ATC cells treated with dual‐drug combination showed evident swelling with characteristic large bubbles from the plasma membrane under bright field (Figure 4D and Videos S1 and S2). Similar morphological changes were not observed in the control group or any single‐drug treatment cells, suggesting dual‐drug combination could induce ATC cells pyroptosis. Transmission electron microscopy showed that the surface of ATC cells treated with apatinib and melittin lacked complex features, and the plasma membrane contained pores and disrupted regions (Figure 4E). In situ live cell staining was performed using Hoechst 33342 and PI to detect the integrity of plasma membrane on ATC cells. The dual‐drug combination group had a higher PI‐positive rate, suggesting more impaired plasma membrane integrity (Figure 4F and Figure S2B). The level of plasma membrane damage was further quantified by LDH release in the supernatant of ATC cells after different treatments. The results showed that the LDH release by ATC cells treated with the dual‐drug combination was significantly increased (Figure 4G). Furthermore, the level of IL‐1β and IL‐18 produced by ATC cells in the dual‐drug combination group was significantly increased both in vitro and in vivo (Figure 4H,I and Figure S3A), suggesting inflammasomes were activated by the combination application of the two drugs.

In mechanism exploration, the increased cleaved caspase‐1 and GSDMD‐N terminal in the dual‐drug combination group were confirmed by Western blotting (Figure 4J). In the meantime, neither activation of caspase‐8 nor phosphorylation of MLKL was observed during this LDH‐releasing process, suggesting that necroptosis was not triggered (Figure S2D). Furthermore, caspase‐4 was not activated during this process as well (Figure S2E). Hence, pyroptosis through the caspase‐1–GSDMD pathway emerged the possible functional role in pyroptosis induction. In apatinib solo treatment, the level of immature pro caspase‐1 was upregulated, but cleaved caspase‐1 did not increase consistently, suggesting the exist of an extra mechanism in caspase‐1 activation by melittin.

**FIGURE 5:**
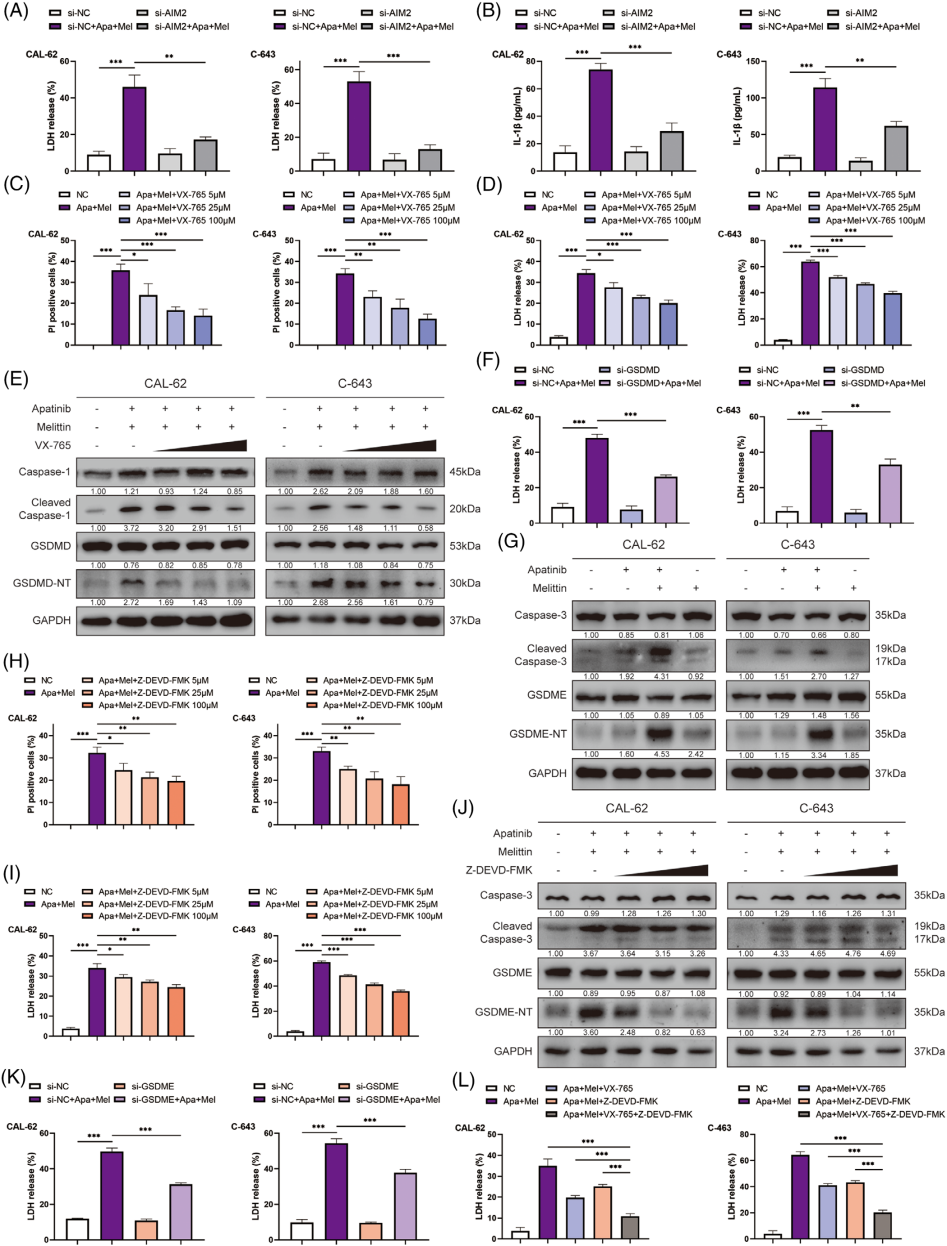
Caspase‐3–GSDME pyroptosis pathway also functioned importantly in addition to caspase‐1–GSDMD pathway. (A and B) LDH release level and IL‐1β production of ATC cells treated by apatinib and melittin with or without AIM2 knocking down. (C and D) Quantitative results of PI‐positive rate and LDH release level of ATC cells treated by apatinib and melittin with ascending concentrations of VX‐765, a specific inhibitor of caspase‐1. (E) VX‐765 reduced the level of GSDMD‐N terminal and cleaved caspase‐1 in apatinib and melittin‐treated ATC cells in a concentration‐dependent manner. (F) LDH release level of ATC cells treated by apatinib and melittin with or without GSDMD knocking down. (G) The protein level of cleaved caspase‐3 and GSDME‐N terminal were also significantly increased when apatinib and melittin were used in combination. (H and I) Quantitative results of PI‐positive rate and LDH release level of ATC cells treated by apatinib and melittin with ascending concentrations of Z‐DEVD‐FMK, a specific inhibitor of caspase‐3. (J) Z‐DEVD‐FMK reduced the level of GSDME‐N terminal in apatinib and melittin‐treated ATC cells in a concentration‐dependent manner. (K) LDH release level of ATC cells treated by apatinib and melittin with or without GSDME knocking down. (L) LDH release level of cells treated by apatinib and melittin with VX‐765 and/or Z‐DEVD‐FMK. Data are represented as mean ± SD; *p < .05, **p < .01, ***p < .001

We speculated that melittin recruits inflammasomes by upregulating the expression of certain NOD‐like receptors, which may result in caspase‐1 activation and massive cleavage of GSDMD (Figure S2C). Therefore, we measured the mRNA transcription levels of nine different NOD‐like receptors by qPCR. mRNA level of AIM2 was most significantly upregulated with the presence of melittin (Figure 4K), which was subsequently confirmed by Western blotting (Figure 4L). In addition, we found that when AIM2 instead of NLRP7 was knocked down via si‐RNA, the pyroptosis of ATC cells was inhibited (Figure 5A,B and Figure S3B–E).

Studies have shown that apatinib can promote the generation of ROS,^28^ and ROS may induce pyroptosis by activating NLRP3.^29^ Therefore, we measured the intracellular ROS levels of ATC cells treated by apatinib and melittin. The results demonstrated that apatinib did promote the generation of ROS significantly, but no apparent pyroptosis could be triggered (Figure S3F). Based on that, the antioxidant N‐Acetyl‐L‐cysteine (NAC) was included to further understand the relationship between ROS production and pyroptosis. The results demonstrated that NAC did not prevent ATC cells from pyroptosis (Figure S3G). Altogether, we confirmed that ROS did not play an important role in the pyroptosis induced by apatinib and melittin.

Thus, melittin promoted the recruitment of pro caspase‐1 through AIM2, which was the key to inducing pyroptosis.

### 3.4 Involvement of caspase‐1–GSDMD pyroptosis pathway was confirmed by specific VX‐765 attenuation

In order to confirm that apatinib combined with melittin caused pyroptosis through the caspase‐1–GSDMD pathway, the specific inhibitor of caspase‐1, VX‐765, was introduced. VX‐765 could reduce the PI‐positive rate as well as the level of LDH release in a concentration‐dependent manner in ATC cells treated with apatinib and melittin (Figure 5C,D and Figure S4A). Western blot also showed that VX‐765 could reduce the level of GSDMD‐N terminal and cleaved caspase‐1 in ATC cells treated by apatinib and melittin in a concentration‐dependent manner (Figure 5E). In addition, knocking down GSDMD via si‐RNA also reduced cell death caused by the dual‐drug combination (Figure 5F), suggesting that GSDMD did play a role in this process. The results confirmed the important role of caspase‐1–GSDMD pyroptosis pathway in extra antitumour effect of the combination of apatinib and melittin. Although VX‐765 can inhibit the pyroptosis caused by the combination of the two drugs by preventing GSDMD from being cleaved, it seems to have only a moderate inhibitory effect, suggesting that there may be other ways to play a role in pyroptosis.

### 3.5 Caspase‐3–GSDME pyroptosis pathway also functioned importantly in addition to caspase‐1–GSDMD pathway

Our previous studies have confirmed that apatinib could induce caspase‐3‐mediated apoptosis by inhibiting ATK–mTOR pathway. Recently, another form of pyroptosis resulting from caspase‐3–GSDME pathway activation has been found.^19^ In Figure 5G, Western blot results showed that cleaved caspase‐3, as well as GSDME‐N terminal, was also intensified in cells treated with apatinib and melittin, suggesting that the caspase‐3–GSDME axis also functioned in addition to caspase‐1–GSDMD pathway activation. Subsequently, Z‐DEVD‐FMK, the caspase‐3‐specific inhibitor, could reduce PI‐positive rate and LDH release level in a concentration‐dependent manner (Figure 5H,I and Figure S4B). In the same manner, Z‐DEVD‐FMK could reduce the level of GSDME‐N terminal (Figure 5J). Furthermore, cell death caused by apatinib and melittin was apparently reduced by knocking down GSDME using si‐RNA (Figure 5K). To confirm the cross‐talk between caspase‐1–GSDMD and caspase‐3–GSDME axes, both VX‐765 and Z‐DEVD‐FMK were included in ATC cell cultures. Our results showed double caspase inhibition could reduce LDH release more than 50% compared with single inhibition (Figure 5L). In addition, the levels of IL‐1β and IL‐18 produced by ATC cells also demonstrated consistent trends (Figure S4C,D). Thus, we identified the important role of caspase‐3–GSDME pyroptosis pathway in addition to caspase‐1–GSDMD pyroptosis pathway in extra antitumour effect of the combination of apatinib and melittin.

### 3.6 A two‐way positive feedback interaction was innovatively proposed between caspase‐1–GSDMD and caspase‐3–GSDME axes

**FIGURE 6:**
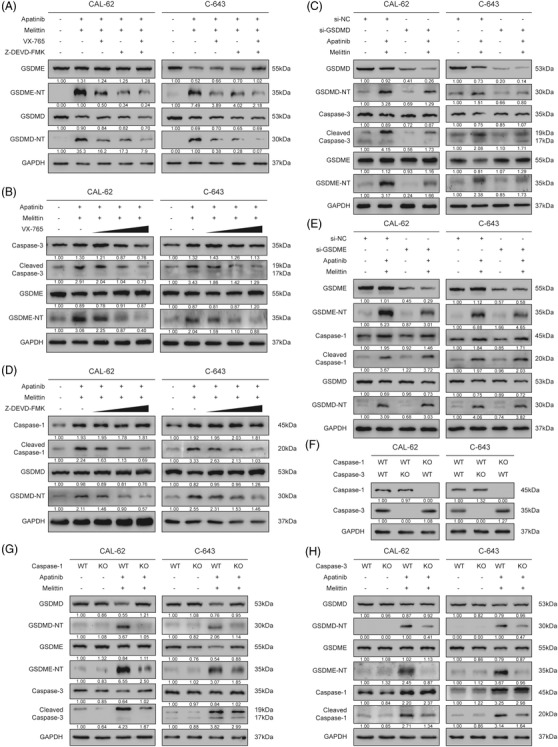
A two‐way positive feedback interaction was innovatively proposed between caspase‐1–GSDMD and caspase‐3–GSDME pathways. (A) VX‐765 could reduce the generation of GSDME‐N terminal, and Z‐DEVD‐FMK could reduce the generation of GSDMD‐N terminal. (B) VX‐765 could reduce the activation of caspase‐3 in a concentration‐dependent manner, resulting in reduced GSDME cleavage. (C) Knocking down of GSDMD reduced the activation of caspase‐3, which led to the reduction of GSDME‐N terminal. (D) Z‐DEVD‐FMK could reduce the activation of caspase‐1 in a concentration‐dependent manner, resulting in reduced GSDMD cleavage. (E) Knocking down of GSDME could not reduce the activation of caspase‐1 as well as GSDMD. (F) Expression of caspase‐1 and caspase‐3 proteins in CAL‐62 and C‐643 knockout clones, or in cells transfected with empty vector. (G) Caspase‐1 knockout reduced both caspase‐3 activation and GSDME cleavage. (H) Caspase‐3 knockout reduced both caspase‐1 activation and GSDMD cleavage

Interestingly, as an inhibitor of caspase‐1, VX‐765 could not only reduce the generation of GSDMD‐N terminal, but also reduce the generation of GSDME‐N terminal (Figure 6A). In mechanism exploration, VX‐765 could reduce the cleavage of caspase‐3 in a concentration‐dependent manner, resulting in reduced GSDME cleavage (Figure 6B). To determine the main molecule (caspase‐1 or GSDMD) that affected activation of caspase‐3, we knocked down GSDMD via si‐RNA. The results showed that knockdown of GSDMD also reduced the activation of caspase‐3, which led to the reduction of GSDME‐N terminal (Figure 6C). These results revealed that GSDMD‐N terminal could affect the activity of caspase‐3 in some way.

Innovatively, as a specific inhibitor of caspase‐3, we found that Z‐DEVD‐FMK could also reduce the cleavage of caspase‐1 and generation of GSDMD‐N terminal in a concentration‐dependent manner (Figure 6A,D). More importantly, knocking down GSDME could not reduce the cleavage of caspase‐1 and the generation of GSDMD‐N terminal (Figure 6E). Thus, it was activated caspase‐3 rather than GSDME that promoted the activation of caspase‐1.

To further confirm the role of caspase‐1 and caspase‐3 in the interaction between two pyroptosis pathways, we established caspase‐1 and caspase‐3 knockout ATC cell line clones, respectively (Figure 6F). The results demonstrated that caspase‐1 knockout reduced both caspase‐3 activation and GSDME cleavage in CAL‐62 and C‐643 cells, whereas caspase‐3 knockout decreased both caspase‐1 activation and GSDMD cleavage (Figure 6G,H).

In view of the fact that cleaved caspase‐1 could affect the activation of caspase‐3 through GSDMD, and cleaved caspase‐3 could enhance the activation of caspase‐1 vice versa, we proposed that there exists a two‐way positive feedback interaction between caspase‐1–GSDMD pathway and caspase‐3–GSDME pathway.

### 3.7 Caspase‐1–GSDMD and caspase‐3–GSDME axes synergistically exerted an antitumour effect in vivo

**FIGURE 7:**
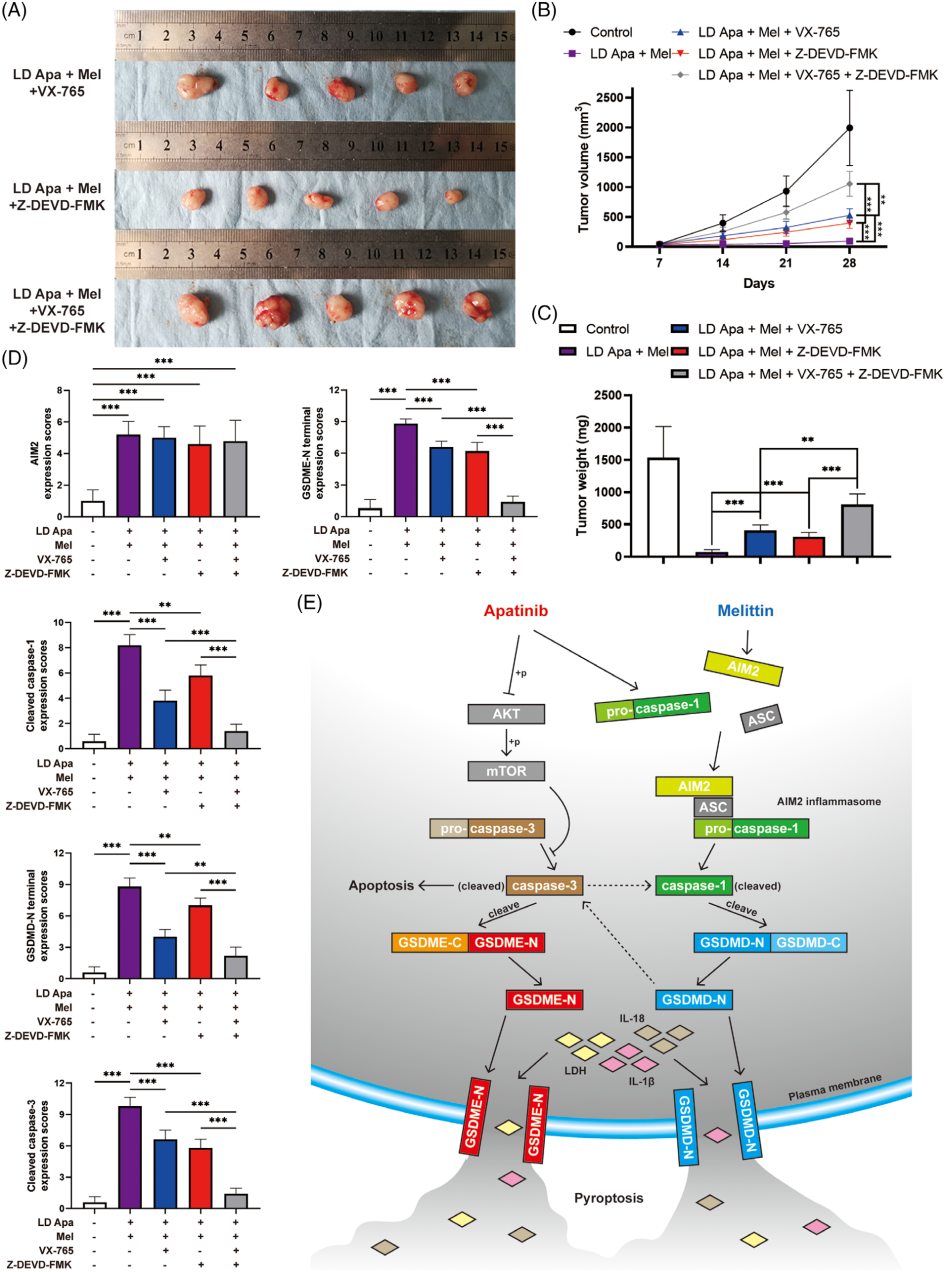
Caspase‐1–GSDMD and caspase‐3–GSDME pathways synergistically exerted an antitumour effect in vivo. (A) Tumours of xenograft mice with subcutaneous injection of CAL‐62 cells. The mice treated with low‐dose apatinib and melittin were given VX‐765, Z‐DEVD‐FMK and both inhibitors, respectively. (B) Tumour volumes were measured every week. (C) Tumour weights were analysed after mice sacrifice. (D) Average scores from IHC staining of AIM2, cleaved caspase‐1, GSDMD‐N terminal, cleaved caspase‐3 and GSDME‐N terminal of tumours after indicated treatment. (E) Schematic representation of a model for the major molecular mechanisms of caspase‐1–GSDMD and caspase‐3–GSDME axes in ATC cells. Data are represented as mean ± SD; *p < .05, **p < .01, ***p < .001

Based on the data above, we investigated the possible synergistical inhibiting effect of VX‐765 and Z‐DEVD‐FMK against the anticancer effect of low‐dose apatinib and melittin in subcutaneous xenograft mice. After establishment of combined treatment models, the mice were simultaneously administered by VX‐765, Z‐DEVD‐FMK and both inhibitors, respectively. Although VX‐765 and Z‐DEVD‐FMK could apparently reverse the antitumour effect of apatinib and melittin by 22.9% and 20.7%, respectively, the synergistical influence on antitumour effect (60.2%) could be identified in the presence of both the inhibitors (Figure 7A–C). In mechanism confirmation, IHC staining showed that VX‐765 reduced not only the positive rate of GSDMD‐N terminal but also cleaved caspase‐3 and GSDME‐N terminal. In the same manner, Z‐DEVD‐FMK reduced the positive rate of GSDME‐N terminal as well as cleaved caspase‐1 and GSDMD‐N terminal (Figure 7D and Figure S5).

Collectively, the two‐way positive feedback interaction was innovatively confirmed between caspase‐1–GSDMD and caspase‐3–GSDME axes in the enhanced antitumour effect of apatinib and melittin.

## 4 DISCUSSION

Currently, effective therapies that can prolong the survival time of ATC patients are still under exploration.^8^, ^30^, ^31^ In our previous study, we confirmed that apatinib has potential therapeutic effects on ATC cells and xenograft model by suppressing angiogenesis and inducing ATC cells apoptosis.^19^, ^32^ We hereby evaluated the therapeutic effect of apatinib on ATC and PDTC through an exploratory, open‐label, nonrandomised, phase II clinical trial. Apatinib demonstrated an inspiring DCR close to 90% after 8 weeks of treatment. Nonetheless, AEs above grade 3 caused an appreciable considerable number of patients to reduce doses or even withdraw from treatment. These AEs are also prevalent in the treatment of other antiangiogenesis targeted agents, such as lenvatinib and sorafenib.^33^, ^34^, ^35^ In view of the promising therapeutic effects of apatinib, it would be more optimal to find ways to reduce AEs and enable better tolerance.

Different from other RCDs, pyroptosis has sophisticated impacts on the microenvironment. Pyroptosis is initially recognised as an original immune reaction to pathogens or its products that occurs in macrophages, monocytes, dendritic cells and T cells.^36^, ^37^ In multiple cancers, pyroptosis has been found activated easily by exogenous agents and demonstrated direct antitumour activity different from apoptosis or autophagy.^7^, ^38^ Furthermore, tumour cells undergoing pyroptosis can recruit tumour‐suppressed immune cells by releasing cytokines and danger‐associated molecular patterns (DAMPs) and trigger subsequent antitumour immune effect.^39^, ^40^, ^41^ Melittin, a water‐soluble peptide separated from honey bee venom, has been applied to alleviate chronic inflammation.^21^ In addition, it has been shown to have cytotoxic effects on many cancer cells due to its lytic activity,^20^, ^21^, ^22^, ^23^, ^42^ and had a high safe dosage in rodent treatment models.^43^ Our results exhibited that in the presence of melittin, apatinib could cause the recruitment and activation of inflammasomes and lead to pyroptosis in a unique way. More importantly, the enhanced antitumour effect of apatinib could be significantly observed both in vitro and in vivo. In this scenario, by activating antitumour pyroptosis, the dosage of antiangiogenesis targeted agents may be decreased without compromising therapeutical benefits, thereby reducing the incidence of AEs.

Although pyroptosis demonstrates a unique antitumour effect in the presence of apatinib and melittin simultaneously, only combination of those two agents can induce pyroptosis in ATC cells. Pyroptosis classically stems from GSDMD proteolytic cleavage as the consequence of inflammatory caspase activation.^1^, ^3^, ^4^ The activation of caspase‐1 inflammasomes as a result of ASC recruitment is often caused by activation of Nod‐like receptors through pyrin domain.^44^ Melittin can cause mitochondrial DNA release into the cytoplasm by destroying the mitochondrial membrane, thereby activating AIM2, the Nod‐like receptor that can bind to cytoplasmic DNA.^45^ Besides, melittin can activate another Nod‐like receptor, NLRP3. However, even with activation of Nod‐like receptors, melittin prevents ATC cells from pyroptosis by suppressing the formation of ASC aggregates and amplification cleavage of caspase‐1.^46^ Our study demonstrated that caspase‐1 could be upregulated by apatinib, and was massively activated in the presence of melittin. Cleavage of GSDMD was subsequently triggered and resulted in pyroptosis within several hours. As this process is less time consuming than caspase‐3‐mediated apoptosis,^6^ pyroptosis caused by apatinib and melittin can be observed earlier than apoptosis. Therefore, we propose that AIM2 activated by melittin and caspase‐1 upregulated by apatinib are both necessary to launch pyroptosis.

Cleaved caspase‐1 has been shown to directly activate Bid–caspase‐9–caspase‐3 axis in the absence of GSDMD, which can be followed by apoptosis and GSDME‐dependent secondary pyroptosis.^47^, ^48^, ^49^ In addition, activated GSDMD could permeabilise the mitochondrial membrane to augment caspase‐3‐mediated apoptosis during caspase‐1 inflammasome activation.^50^ Our results showed that inhibiting the activity of caspase‐1 or knocking down GSDMD could reduce the activation of caspase‐3 and the production of GSDME‐NT, which is consistent with previous reports. However, in this study, activated caspase‐3 cannot only cleave GSDME to further boost the pyroptosis process, but in turn enhance the activation of caspase‐1. This positive stimulation effect of cleaved caspase‐3 on the caspase‐1–GSDMD pathway has not been reported before. In this scenario, cleaved caspase‐1 activates caspase‐3 through GSDMD, and cleaved caspase‐3 intensifies the activation of caspase‐1 vice versa. These processes constitute a unique system involving a two‐way positive feedback regulation between caspase‐1–GSDMD and caspase‐3–GSDME axes. More importantly, this positive feedback regulation may improve therapeutic effect of antiangiogenesis targeted agents, and provide a new prospect of targeted therapy.

Recent studies using next‐generation sequencing technology have decoded the molecular pathogenesis of thyroid cancer developments.^51^, ^52^ Compared to DTCs, ATCs have a higher frequency of mutations in RAF, RAS and TERT promoter. The cell line CAL‐62 used in this study carries KRAS mutation, whereas C‐643 carries HRAS and TERT promoter mutations. These mutations function importantly in pyroptosis evasion by activating survival‐promoting signalling pathway, which are closely related to tumour occurrence and development.^15^, ^16^ Therefore, our findings suggest that pyroptosis regulation could be a novel promising therapeutical target for cancer. Antitumour effect mediated by pyroptosis will provide a new prospect of targeted therapy.

## 5 CONCLUSION

In summary, apatinib demonstrated a promising therapeutic benefit in anaplastic or poorly differentiated thyroid carcinoma by a DCR of 88.2%. However, treatment was terminated in 23.5% of the patients due to intolerable toxicity. The consistent data from in vitro and in vivo studies concluded that low‐dosage apatinib with melittin could synergistically achieve a comparable therapeutic potential, which could reduce AEs. Furthermore, a novel antitumour effect of pyroptosis induced by combination of apatinib and melittin in ATC was identified. AIM2 activated by melittin and caspase‐1 upregulated by apatinib are both necessary to launch pyroptosis. Mechanistically, both caspase‐1–GSDMD and caspase‐3–GSDME pyroptosis pathways were the key to this extra antitumour effect. More importantly, cleaved caspase‐1 activated caspase‐3 through generating GSDMD‐N terminal, and cleaved caspase‐3 intensified the activation of caspase‐1 vice versa. These processes constitute a unique system involving a two‐way positive feedback regulation between caspase‐1–GSDMD and caspase‐3–GSDME axes. This positive feedback regulation may improve therapeutic effect of antiangiogenesis targeted agents, and provide a new prospect of targeted therapy.

## CONFLICT OF INTEREST

The authors declare that there is no conflict of interest.

## Supporting information


**Table S1** Primers for qRT‐PCR
**Table S2** Combination index (CI) data for the combination of apatinib and melittin using the Chou–Talalay method
**Figure S1** Apatinib and melittin had a synergistic effect on the treatment of ATC in vitro. (A) Dose–effect curve of indicated treatment. (B) Median effect plot of indicated treatment. (C) Isobologram at *F*_a _= 0.5, 0.75 and 0.9 for combined application of apatinib and melittin. (D) *F*_a_‐dose reduction index (DRI) plot for combined application of apatinib and melittin
**Figure S2** Pyroptosis rather than necroptosis provided an extra antitumour effect of the combination of apatinib and melittin. (A) Directed acyclic graph of GO molecular function enrichment using RNA‐seq data. (B) Representative images of in situ Hoechst 33342/PI double staining of cells after indicated treatment. (C) Nine Nod‐like receptors in the KEGG signalling pathway map. (D) The combination of apatinib and melittin could not cause the activation of caspase‐8 and the phosphorylation of MLKL. (E) Apatinib and melittin could not cause the activation of caspase‐4
**Figure S3** Apatinib and melittin induced ATC cell pyroptosis through AIM2–caspase‐1 axis. (A) Representative images of IHC staining of IL‐1β and IL‐18 of xenograft models (scale bar: 50 μm). (B and C) mRNA and protein level of AIM2 and NLRP7 in ATC cells transfected with si‐RNA targeting AIM2 or NLRP7, or in cells transfected with nontargeting si‐RNA. (D and E) LDH release level and IL‐1β production of ATC cells treated by apatinib and melittin with or without NLRP7 knocking down. (F) Apatinib upregulated the intracellular ROS level of ATC cells, whereas melittin had no significant effect on ROS. (G) LDH release level of ATC cells treated by apatinib and melittin with or without 2 mM NAC. Data are represented as mean ± SD; *p < .05, **p < .01, ***p < .001
**Figure S4** Pyroptosis of ATC cells could be inhibited by VX‐765 and Z‐DEVD‐FMK. (A) VX‐765 reduced the PI‐positive rate of ATC cells treated by apatinib and melittin in a concentration‐dependent manner. (B) Z‐DEVD‐FMK reduced the PI‐positive rate of ATC cells treated by apatinib and melittin in a concentration‐dependent manner. (C and D) IL‐1β and IL‐18 production of ATC cells treated by apatinib and melittin with VX‐765 and/or Z‐DEVD‐FMK. Data are represented as mean ± SD; *p < .05, **p < .01, ***p < .001
**Figure S5** Representative images of IHC staining of tumours of indicated treatment (scale bar: 50 μm)
**Video S1** Morphological change of CAL‐62 cells after treatment with apatinib and melittin
**Video S2** Morphological change of C‐643 cells after treatment with apatinib and melittin

Figure S1

Figure S2

Figure S3

Figure S4

Figure S5

C‐643 CASP1 KO DNA sequencing results surrounding sgRNA targets

C‐643 CASP3 KO DNA sequencing results surrounding sgRNA targets

CAL‐62 CASP1 KO DNA sequencing results surrounding sgRNA targets

CAL‐62 CASP3 KO DNA sequencing results surrounding sgRNA targets

Video S1

Video S2
