# Supplementary material for: The central role of a two‐way positive feedback pathway in molecular targeted therapies‐mediated pyroptosis in anaplastic thyroid cancer
Source: Clin Transl Med. 2022 Feb 20;12(2):e727. doi: 10.1002/ctm2.727 (PMC8858618; doi:10.1002/ctm2.727)
Supplement: Supplementary file 1 — Table S1 Primers for qRT‐PCR Table S2 Combination index (CI) data for the combination of apatinib and melittin using the Chou–Talalay method Figure S1 Apatinib and melittin had a synergistic effect on the treatment of ATC in vitro. (A) Dose–effect curve of indicated treatment. (B) Median effect plot of indicated treatment. (C) Isobologram at F a = 0.5, 0.75 and 0.9 for combined application of apatinib and melittin. (D) F a‐dose reduction index (DRI) plot for combined application of apatinib and melittin Figure S2 Pyroptosis rather than necroptosis provided an extra antitumour effect of the combination of apatinib and melittin. (A) Directed acyclic graph of GO molecular function enrichment using RNA‐seq data. (B) Representative images of in situ Hoechst 33342/PI double staining of cells after indicated treatment. (C) Nine Nod‐like receptors in the KEGG signalling pathway map. (D) The combination of apatinib and melittin could not cause the activation of caspase‐8 and the phosphorylation of MLKL. (E) Apatinib and melittin could not cause the activation of caspase‐4 Figure S3 Apatinib and melittin induced ATC cell pyroptosis through AIM2–caspase‐1 axis. (A) Representative images of IHC staining of IL‐1β and IL‐18 of xenograft models (scale bar: 50 μm). (B and C) mRNA and protein level of AIM2 and NLRP7 in ATC cells transfected with si‐RNA targeting AIM2 or NLRP7, or in cells transfected with nontargeting si‐RNA. (D and E) LDH release level and IL‐1β production of ATC cells treated by apatinib and melittin with or without NLRP7 knocking down. (F) Apatinib upregulated the intracellular ROS level of ATC cells, whereas melittin had no significant effect on ROS. (G) LDH release level of ATC cells treated by apatinib and melittin with or without 2 mM NAC. Data are represented as mean ± SD; *p < .05, **p < .01, ***p < .001 Figure S4 Pyroptosis of ATC cells could be inhibited by VX‐765 and Z‐DEVD‐FMK. (A) VX‐765 reduced the PI‐positive rate of ATC cells treated by apatinib and [file CTM2-12-e727-s003.docx]

**Table S1** Primers for qRT-PCR

| Gene | Sequence |
| --- | --- |
| NLRP1 | Forward primer: CCACAACCCTCTGTCTACATTAC |
|  | Reverse primer: GCCCCATCTAACCCATGCTTC |
| NLRP3 | Forward primer: CGTGAGTCCCATTAAGATGGAGT |
|  | Reverse primer: CCCGACAGTGGATATAGAACAGA |
| NLRP6 | Forward primer: TTCGGCTGCATGGTTTCAGAG |
|  | Reverse primer: CGTCTCGTACAGGCAGTACAG |
| NLRP7 | Forward primer: CTAAAACACCTACGCCTCTGGA |
|  | Reverse primer: CCAGGTCCAGAGTTTCAAGC |
| NLRP12 | Forward primer: TGAGTTTCAACGACCTGGGAG |
|  | Reverse primer: CCACAGCTATCCAGCCACAGT |
| NLRC4 | Forward primer: TGCATCATTGAAGGGGAATCTG |
|  | Reverse primer: GATTGTGCCAGGTATATCCAGG |
| AIM2 | Forward primer: GTAGTCCAGAAGGTAACAGAA |
|  | Reverse primer: CTTAGACCAGTTGGCTTGA |
| MEFV | Forward primer: TAAGACCCCTAGTGACCATCTG |
|  | Reverse primer: TTCCCCATAGTAGGTGACCAG |
| IFI16 | Forward primer: AGCAGAATAGGAGCAAGCCA |
|  | Reverse primer: CGGAACCGCAGGATGTTGTA |

**Table S2** CI data for the combination of apatinib and melittin using the Chou-Talalay method

CAL-62

|  | CI values at: | | | |
| --- | --- | --- | --- | --- |
| Combo | ED50 | ED75 | ED90 | ED95 |
| A+M | 0.74613 | 0.72331 | 0.71005 | 0.70583 |

Data for Fa = 0.5

| Drug/Combo | CI value | Dose Apa | Dose Mel |
| --- | --- | --- | --- |
| Apa |  | 68.4115 |  |
| Mel |  |  | 8.69247 |
| A+M | 0.74613 | 19.8303 | 3.96605 |

Data for Fa = 0.75

| Drug/Combo | CI value | Dose Apa | Dose Mel |
| --- | --- | --- | --- |
| Apa |  | 137.845 |  |
| Mel |  |  | 13.8069 |
| A+M | 0.72331 | 33.2710 | 6.65419 |

Data for Fa = 0.9

| Drug/Combo | CI value | Dose Apa | Dose Mel |
| --- | --- | --- | --- |
| Apa |  | 277.750 |  |
| Mel |  |  | 21.9307 |
| A+M | 0.71005 | 55.8215 | 11.1643 |

Data for Fa = 0.95

| Drug/Combo | CI value | Dose Apa | Dose Mel |
| --- | --- | --- | --- |
| Apa |  | 447.298 |  |
| Mel |  |  | 30.0421 |
| A+M | 0.70583 | 79.3697 | 15.8739 |

C-643

|  | CI values at: | | | |
| --- | --- | --- | --- | --- |
| Combo | ED50 | ED75 | ED90 | ED95 |
| A+M | 0.74633 | 0.79053 | 0.84094 | 0.87913 |

Data for Fa = 0.5

| Drug/Combo | CI value | Dose Apa | Dose Mel |
| --- | --- | --- | --- |
| Apa |  | 76.8012 |  |
| Mel |  |  | 2.71082 |
| A+M | 0.74633 | 23.7192 | 1.18596 |

Data for Fa = 0.75

| Drug/Combo | CI value | Dose Apa | Dose Mel |
| --- | --- | --- | --- |
| Apa |  | 128.576 |  |
| Mel |  |  | 3.96641 |
| A+M | 0.79053 | 38.7832 | 1.93916 |

Data for Fa = 0.9

| Drug/Combo | CI value | Dose Apa | Dose Mel |
| --- | --- | --- | --- |
| Apa |  | 215.254 |  |
| Mel |  |  | 5.80355 |
| A+M | 0.84094 | 63.4143 | 3.17072 |

Data for Fa = 0.95

| Drug/Combo | CI value | Dose Apa | Dose Mel |
| --- | --- | --- | --- |
| Apa |  | 305.606 |  |
| Mel |  |  | 7.51829 |
| A+M | 0.87913 | 88.5988 | 4.42994 |

**SUPPLEMENTARY INFORMATION**

**Figure S1** Apatinib and melittin had a synergistic effect on the treatment of ATC *in vitro*. (A) Dose-effect curve of indicated treatment. (B) Median-effect plot of indicated treatment. (C) Isobologram at F_a_=0.5, F_a_=0.75, and F_a_=0.9 for combined application of apatinib and melittin. (D) F_a_-Dose-reduction index (DRI) plot for combined application of apatinib and melittin.

**Figure S2** Pyroptosis rather than necroptosis provided an extra anti-tumor effect of the combination of apatinib and melittin. (A) Directed acyclic graph of GO molecular function enrichment using RNA-seq data. (B) Representative images of *in situ* Hoechst 33342/PI double staining of cells after indicated treatment. (C) Nine Nod-like receptors in the KEGG signaling pathway map. (D) The combination of apatinib and melittin could not cause the activation of caspase-8 and the phosphorylation of MLKL. (E) Apatinib and melittin could not cause the activation of caspase-4.

**Figure S3** Apatinib and melittin induced ATC cell pyroptosis through AIM2-caspase-1 axis. (A) Representative images of IHC staining of IL-1β and IL-18 of xenograft models (scale bar, 50μm). (B, C) mRNA and protein level of AIM2 and NLRP7 in ATC cells transfected with si-RNA targeting AIM2 or NLRP7, or in cells transfected with nontargeting si-RNA. (D, E) LDH release level and IL-1β production of ATC cells treated by apatinib and melittin with or without NLRP7 knocking-down. (F) Apatinib up-regulated the intracellular ROS level of ATC cells, while melittin had no significant effect on ROS. (G) LDH release level of ATC cells treated by apatinib and melittin with or without 2mM NAC. Data are represented as mean ± SD, **p* < 0.05, ***p* < 0.01, ****p* < 0.001.

**Figure S4** Pyroptosis of ATC cells could be inhibited by VX-765 and Z-DEVD-FMK. (A) VX-765 reduced the PI positive rate of ATC cells treated by apatinib and melittin in a concentration-dependent manner. (B) Z-DEVD-FMK reduced the PI positive rate of ATC cells treated by apatinib and melittin in a concentration-dependent manner. (C, D) IL-1β and IL-18 production of ATC cells treated by apatinib and melittin with VX-765 and/or Z-DEVD-FMK. Data are represented as mean ± SD, **p* < 0.05, ***p* < 0.01, ****p* < 0.001.

**Figure S5** Representative images of IHC staining of tumors of indicated treatment (scale bar, 50μm).
