# Supplementary figures and images for: The central role of a two‐way positive feedback pathway in molecular targeted therapies‐mediated pyroptosis in anaplastic thyroid cancer
Source: Clin Transl Med. 2022 Feb 20;12(2):e727. doi: 10.1002/ctm2.727 (PMC8858618; doi:10.1002/ctm2.727)

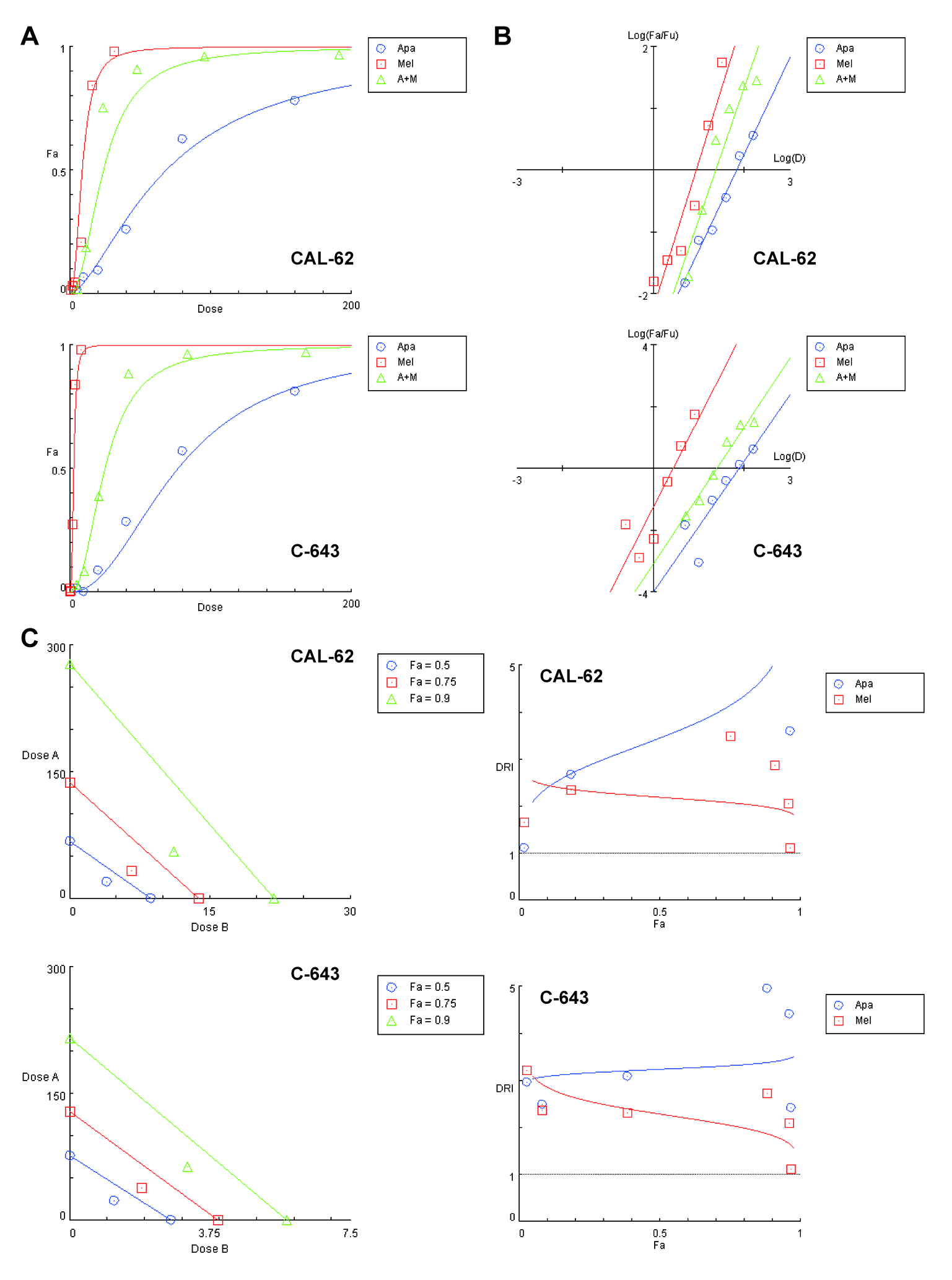

Supplement: Supplementary file 2 — Figure S1 [file CTM2-12-e727-s012.tif]

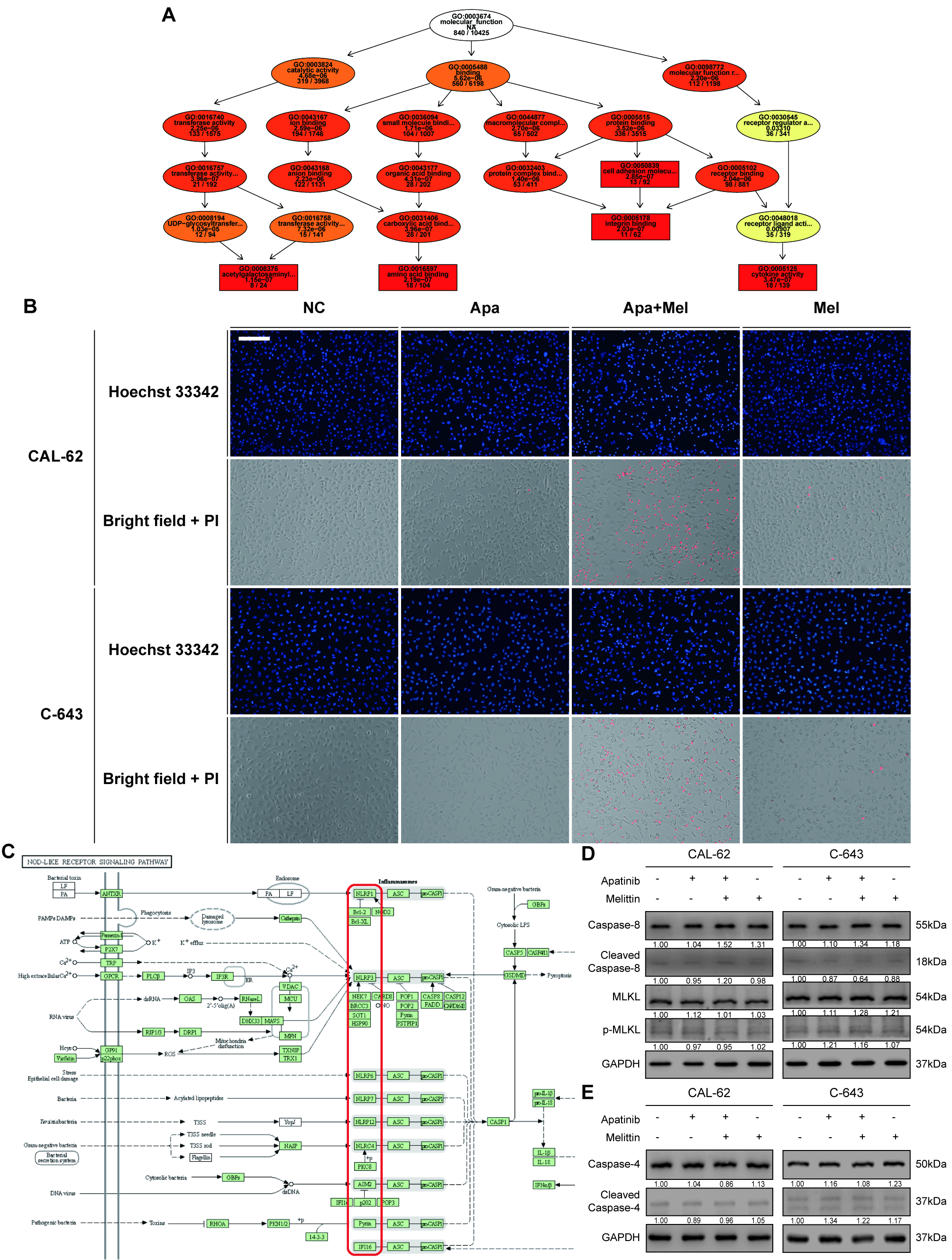

Supplement: Supplementary file 3 — Figure S2 [file CTM2-12-e727-s008.tif]

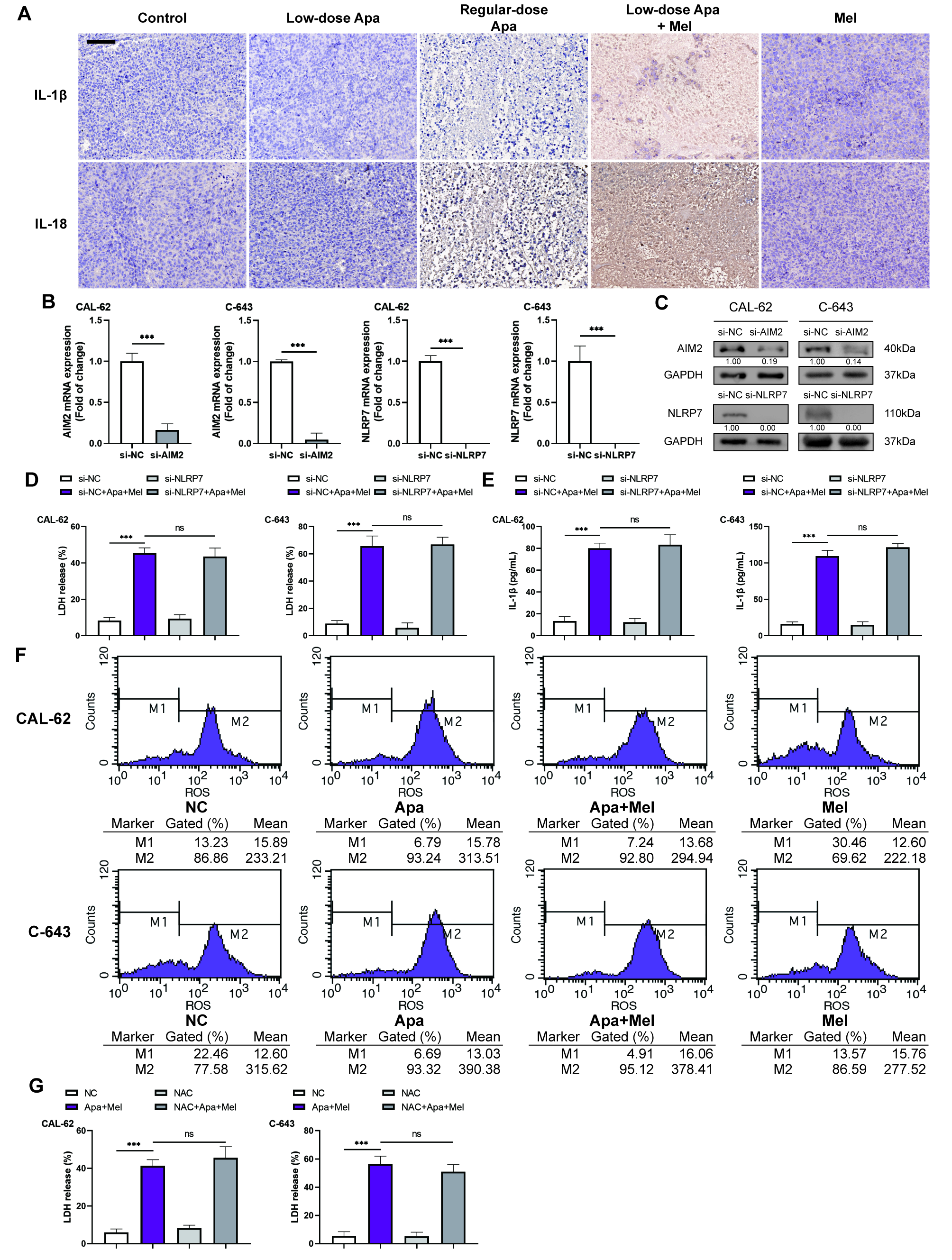

Supplement: Supplementary file 4 — Figure S3 [file CTM2-12-e727-s005.tif]

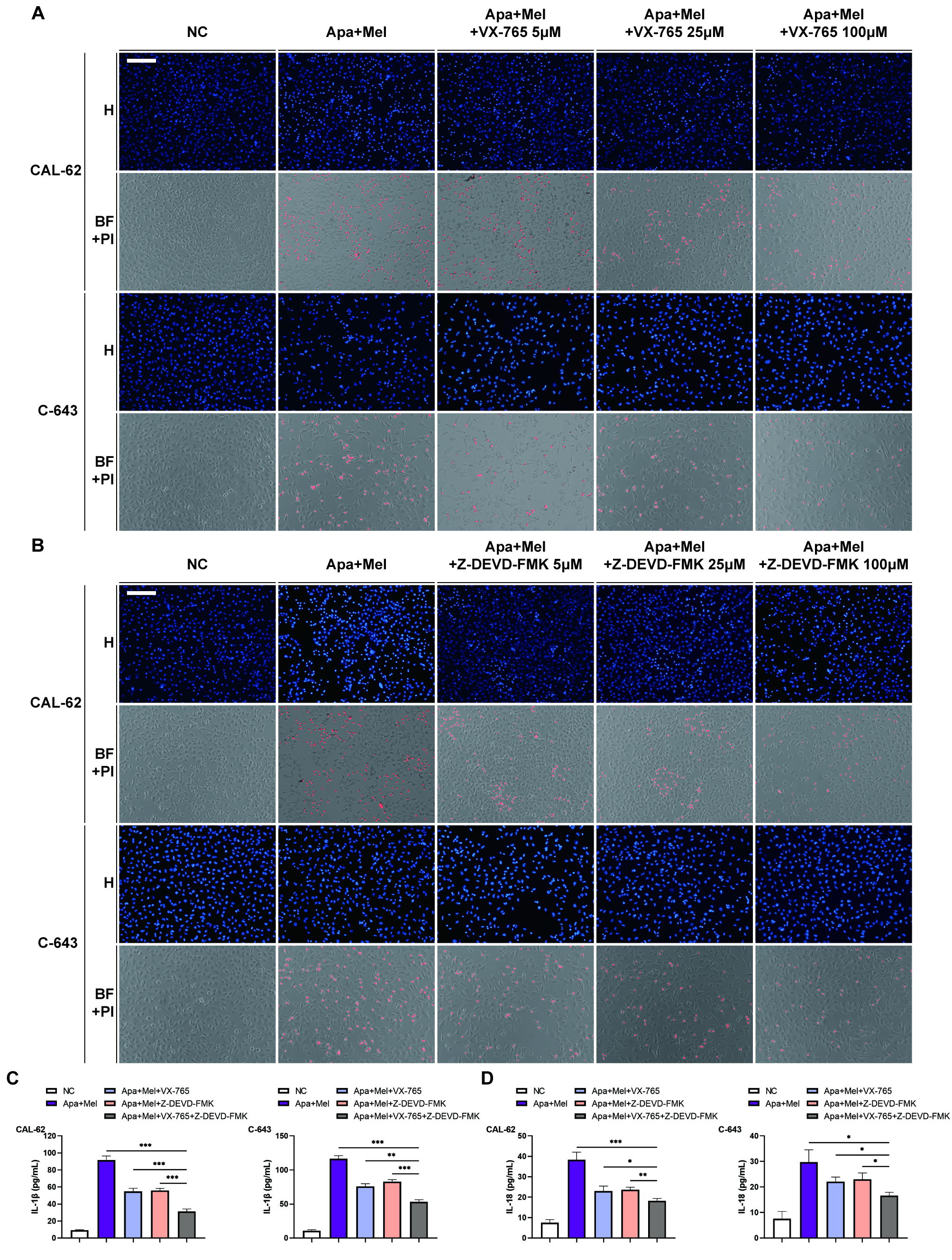

Supplement: Supplementary file 5 — Figure S4 [file CTM2-12-e727-s004.tif]

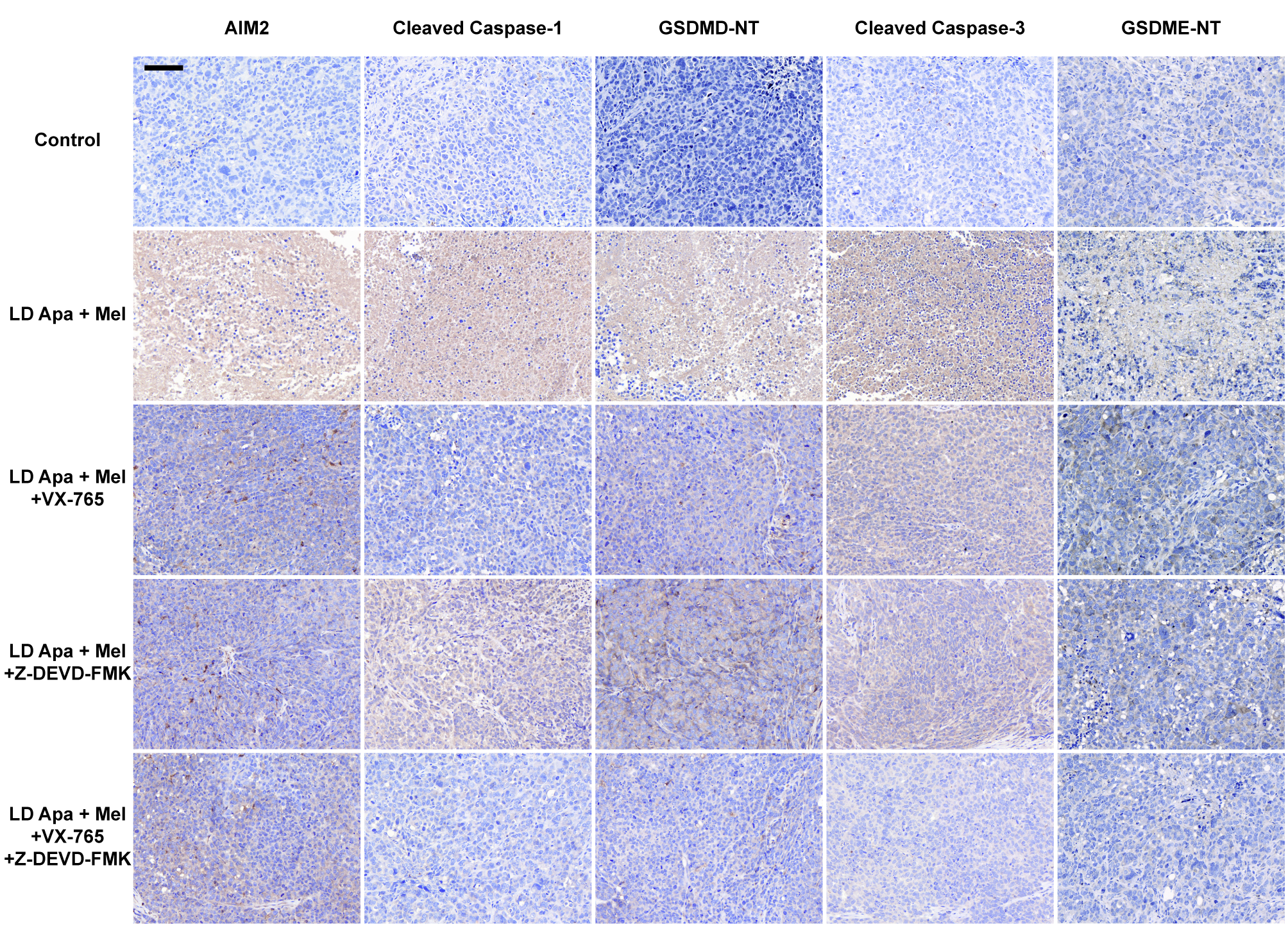

Supplement: Supplementary file 6 — Figure S5 [file CTM2-12-e727-s009.tif]
